# Breed and ruminal fraction effects on bacterial and archaeal community composition in sheep

**DOI:** 10.1038/s41598-023-28909-1

**Published:** 2023-02-27

**Authors:** Steven McLoughlin, Charles Spillane, Francis P. Campion, Noel Claffey, Chrystian C. Sosa, Yvonne McNicholas, Paul E. Smith, Michael G. Diskin, Sinéad M. Waters

**Affiliations:** 1 Animal and Bioscience Research Department, Animal and Grassland Research and Innovation Centre, Teagasc, Athenry, Co. Galway H65 R718 Ireland; 2 Genetics and Biotechnology Laboratory, Agriculture & Bioeconomy Research Centre (ABC), Ryan Institute, University of Galway, University Road, Galway, H91 REW4 Ireland

**Keywords:** Environmental impact, Microbial ecology

## Abstract

While the breed of cattle can impact on the composition and structure of microbial communities in the rumen, breed-specific effects on rumen microbial communities have rarely been examined in sheep. In addition, rumen microbial composition can differ between ruminal fractions, and be associated with ruminant feed efficiency and methane emissions. In this study, 16S rRNA amplicon sequencing was used to investigate the effects of breed and ruminal fraction on bacterial and archaeal communities in sheep. Solid, liquid and epithelial rumen samples were obtained from a total of 36 lambs, across 4 different sheep breeds (Cheviot (n = 10), Connemara (n = 6), Lanark (n = 10) and Perth (n = 10)), undergoing detailed measurements of feed efficiency, who were offered a nut based cereal diet *ad-libitum* supplemented with grass silage. Our results demonstrate that the feed conversion ratio (FCR) was lowest for the Cheviot (most efficient), and highest for the Connemara breed (least efficient). In the solid fraction, bacterial community richness was lowest in the Cheviot breed, while *Sharpea azabuensis* was most abundant in the Perth breed. Lanark, Cheviot and Perth breeds exhibited a significantly higher abundance of epithelial associated *Succiniclasticum* compared to the Connemara breed. When comparing ruminal fractions, *Campylobacter*, Family XIII, *Mogibacterium*, and Lachnospiraceae *UCG-008* were most abundant in the epithelial fraction. Our findings indicate that breed can impact the abundance of specific bacterial taxa in sheep while having little effect on the overall composition of the microbial community. This finding has implications for genetic selection breeding programs aimed at improving feed conversion efficiency of sheep. Furthermore, the variations in the distribution of bacterial species identified between ruminal fractions, notably between solid and epithelial fractions, reveals a rumen fraction bias, which has implications for sheep rumen sampling techniques.

## Introduction

Ruminant livestock contribute significantly to food security by converting human indigestible plant matter, into high quality sources of dairy and meat proteins, for human consumption^1^. A sustainable supply of animal derived protein over the next decades will be key to meeting the nutritional requirements of an estimated nine billion people by 2050^2^. However, livestock production systems are also a major source of anthropogenic greenhouse gas emissions with enteric fermentation estimated to contribute to 35–40% of global methane emissions^3^. As a result, there is an urgent need to increase animal protein production to fulfil nutritional demand while simultaneously improving the livestock industry's environmental sustainability metrics. Increasing the feed conversion efficiency of livestock is proposed as a mitigation solution for the livestock industry, as more feed efficient ruminants emit less methane than their less efficient counterparts^4–6^. In addition, improvements to feed efficiency are likely to benefit farm profitability^7^ while reducing the quantity of global land dedicated to producing feed for the livestock industry^8^.

Mountain or hill sheep production is a significant agricultural enterprise that provides social and economic health in rural areas across the globe, while also protecting natural habitats and promoting biodiversity^9^. In Ireland and the UK, popular hill sheep breeds include the Scottish Blackface (SB) and the Cheviot. SB are mountain breeds which display adaptive tolerance to harsh environmental conditions and challenging terrains with low-energy vegetation^10^. The wide distribution of SB breeds across the UK and Ireland has led to evolutionary changes within the breed, influenced largely by environmental pressures between different habitats^10^. As a result, a range of different strains of SB breed exist today including the Lanark, Perth and Connemara breed types which all vary in body and wool composition. The Cheviot breed is also well-suited to highland farming, and while not as resilient as the SB^11^, they are slightly larger and produce lambs that mature quickly^12^.

Sheep, like all ruminants, rely on a complex and dynamic microbial ecosystem (anaerobic bacteria, archaea, fungi and protozoa) within their rumen to derive energy from feed^13^. The rumen is composed of three environmental niches, namely the solid-, liquid-, and epithelial-fractions^14–16^. The solid fraction, comprised of ingested feed, is primarily colonised by feed adherent microbes that breakdown fibrous matter^14^. The liquid fraction consists of the fluid within the rumen and provides an environment for free living microbes involved in the metabolism of soluble nutrients^17^. Finally, the epithelial fraction refers to the epithelial lining of the rumen, which harbours microbes active in tissue recycling^18^, oxygen scavenging^19^, and urea hydrolysis^20^ and is critically important for bioconversions and nutrient uptake as the cellular interface with the host animal. Research in both bovine and ovine models have reported differences in the microbial taxonomic profiles between ruminal fractions^14,16^ with the epithelial being mostly distinct from the solid and liquid fractions, whereas the solid and liquid fractions tend to be more similar^17,21^. To date, most studies investigating breed effects have been conducted using the rumen digesta samples with minimal exploration of the microbial community associated with the epithelial fraction.

Research has revealed links between the rumen microbiota, feed efficiency and methane emissions in both cattle and sheep, with differences in microbial diversity and abundances between divergent animal cohorts^21–24^. Understanding factors that influence the composition and diversity of the rumen microbiome is critical for improving strategies to enhance feed efficiency and reduce ruminant methane emissions. Recently, studies in cattle have shown that microbial taxonomic profiles differ between breeds^25,26^, suggesting that host genetics may regulate the composition of the rumen microbiome. However, such effects have not been explored in sheep.

It is unclear whether breed specific findings in cattle can be translated to sheep. Taxa-specific research is imperative given the importance of the global sheep industry from environmental, economic and social perspectives. In addition, while cattle studies have provided some indication that breed plays an important role in shaping the rumen microbiome, to date, such effects have not been investigated across all three ruminal fractions. Hence, the objectives of the current study are twofold. Firstly, to investigate the effect of breed on bacterial and archaeal populations in the solid, liquid and epithelial rumen fractions of sheep, and secondly to investigate the effect of the ruminal fraction on the microbial populations in each of the breeds, using 16S rRNA amplicon sequencing.

## Results

### Breed differences in animal feed conversion and economic trait performance

Throughout the feed intake measurement period, summary statistics shows animals on test had an average DMI of 1.11 kg/d (SD = 0.18), ADG of 0.27 kg/d (SD = 0.1), FCR of 4.04 kg of DMI/ Kg of ADG (SD = 0.1), start weight of 29.60 kg (SD = 3.7), final live weight of 46.00 kg (SD = 2.9), carcass weight of 20.20 kg (SD = 1.6), and a KO% of 44.1% (SD = 2.3). Average daily gain (*P* = 0.005), FCR (*P* = 0.035), CW (*P* < 0.04) and start weight (*P* < 0.036) were all significantly affected by breed. Summary statistics, along with comparisons amongst breeds for animal performance, feed intake and feed efficiency are displayed in Table 1. In summary, the Cheviot breed had the lowest FCR and the highest ADG, carcass weight, and start weight among all breeds, with differences in ADG and FCR being significant when compared to the Connemara breed and differences in carcass and start weight being significant when compared to the Lanark breed. In addition, the Cheviot breed had the fastest maturing lambs with 80% of lambs reaching maturity within the first 42 days (data not shown) and a mean LW of 47.1 kg (Table 1).

**Table 1:** Animal production traits for Cheviot, Connemara, Lanark, and Perth.

	Cheviot Mean ± sd	Connemara Mean ± sd	Lanark Mean ± sd	Perth Mean ± sd	Anova Pvalue	Overall Mean ± sd
ADG (kg/d)	0.3 ± 0.06^a^	0.2 ± 0.06^b^	0.3 ± 0.08^ab^	0.3 ± 0.05^a^	0.005	0.27 ± 0.1
DMI (kg/d)	1.151 ± 0.21	1.125 ± 0.16	1.090 ± 0.17	1.086 ± 0.17	0.843	1.113 ± 0.18
FCR (DMI/ADG)	3.7 ± 0.54^b^	5.1 ± 1.46^a^	4.1 ± 0.95^ab^	3.8 ± 0.75^ab^	0.035	4.04 ± 0.1
LW (kg)	47.1 ± 3.37	45.6 ± 2.84	44.5 ± 1.47	46.8 ± 3.17	0.161	46.0 ± 2.9
CW (kg)	21.3 ± 1.73^a^	20.2 ± 0.65^ab^	19.4 ± 0.88^b^	19.9 ± 1.86^ab^	0.040	20.2 ± 1.6
LW gain (kg)	15.1 ± 4.19	14.3 ± 6.35	14.5 ± 5.16	16.4 ± 4.47	0.717	15.2 ± 4.8
Start weight (kg)	31.6 ± 3.88^a^	29.2 ± 3.19^ab^	27.7 ± 2.28^b^	29.3 ± 4.10^ab^	0.036	29.6 ± 3.7
KO%	45.4 ± 2.19	44.5 ± 2.82	43.7 ± 1.88	42.7 ± 1.79	0.072	44.1 ± 2.3

Mean ± Sd, ANOVA P value and Tukey HSD pairwise comparisons (superscripts) presented in table. ADG (Average Daily Gain), DMI (Dry Matter Intake) FCR (Feed Conversion Ratio) LW (Live Weight) CW (Carcass Weight) LW Gain (Live Weight Gain) and kill out percentage (KO%).

### Overall microbial community structure

After data processing, filtering, and removal of chimeras and lowly sequenced samples a total of 5,411,353 reads remained, with an average of 91.3% of reads surviving. The average number of reads per sample was 64,420, which mapped to 2547 ASVs. After removal of taxa unassigned at the phylum level 2434 ASV’s remained. Analysis of the ASVs across all samples revealed that bacteria and archaea represented 95.4 and 4.6% of the microbial population, respectively. A total of 19 bacterial taxa were classified at the phylum level, with Firmicutes being the most abundant (45.8%), followed by Bacteroidetes (33.4%) and Proteobacteria (8.0%). There were 192 taxa classified at the genus level, with *Prevotella_1* (13.1%) and *Prevotella_7* (13.1%) being the most dominant followed by *Succinivibrio* (6.3%). Methanobrevibacter was shown to be the most abundant archaeal genus. (78.1%). In this study, no non-methanogenic archaeal taxa were identified.

### Breed effects on bacterial and archaeal populations in the solid ruminal fraction

**Figure 1:**
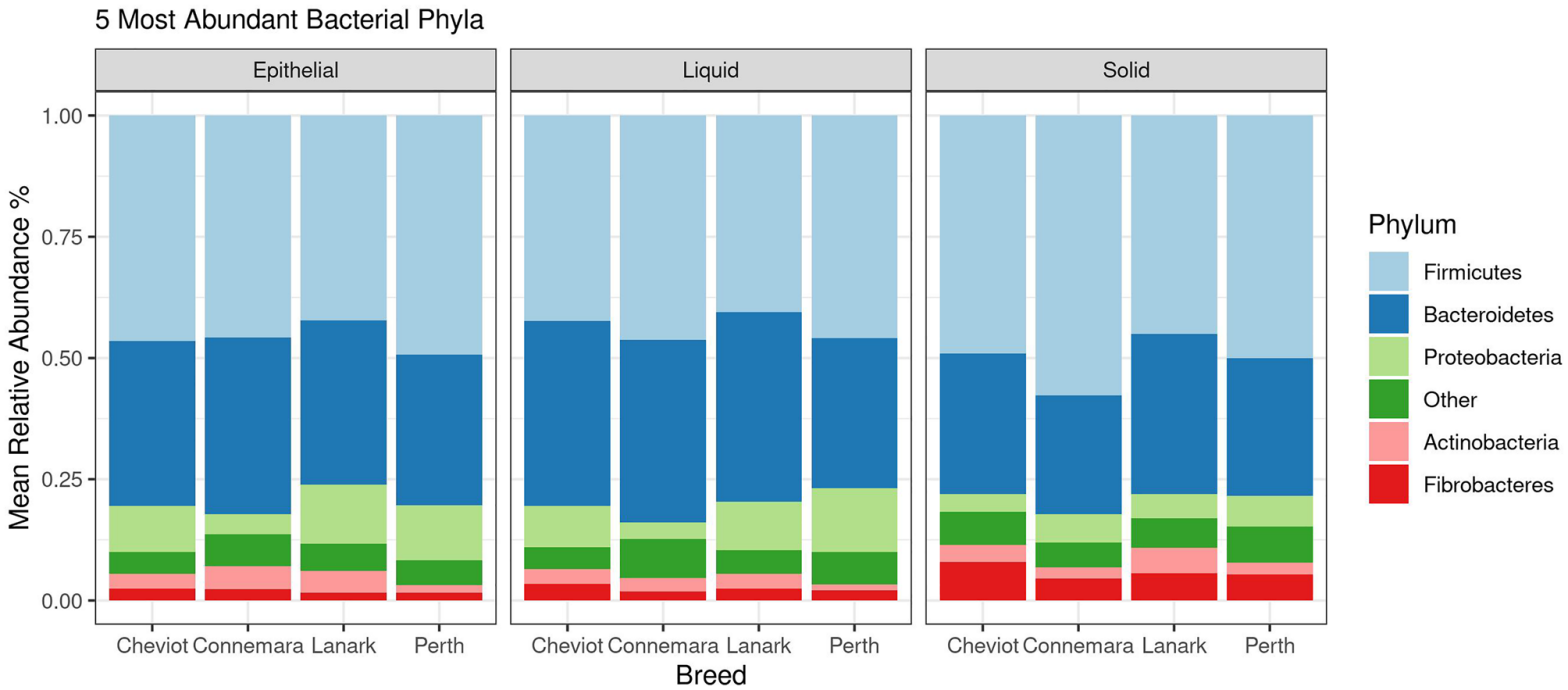
Stack barchart representing the mean relative abundance of the 5 most dominant phyla across breeds (i.e. Cheviot, Connemara, Lanark, Perth) for solid liquid and epithelial ruminal fractions. Solid (Cheviot *n* = 8, Connemara *n* = 5, Lanark *n* = 9, Perth *n* = 7), liquid (Cheviot *n* = 9, Connemara *n* = 5, Lanark *n* = 9, Perth *n* = 5), epithelial (Cheviot *n* = 9, Connemara *n* = 3, Lanark *n* = 6, Perth *n* = 8).

**Figure 2:**
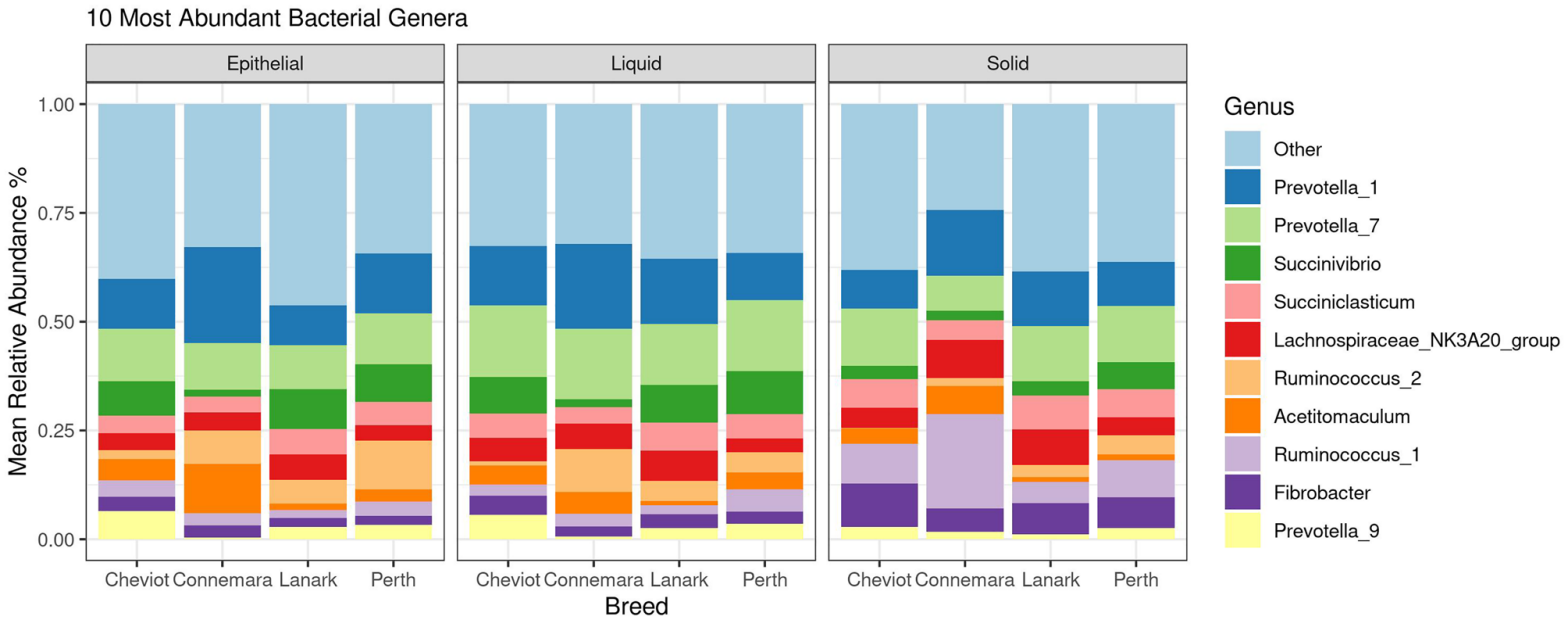
Stack barchart representing the mean relative abundance of the 10 most dominant genera across breeds (i.e. Cheviot, Connemara, Lanark, Perth) for solid liquid and epithelial ruminal fractions. Solid (Cheviot *n* = 8, Connemara *n* = 5, Lanark *n* = 9, Perth *n* = 7), liquid (Cheviot *n* = 9, Connemara *n* = 5, Lanark *n* = 9, Perth *n* = 5), epithelial (Cheviot *n* = 9, Connemara *n* = 3, Lanark *n* = 6, Perth *n* = 8).

In the solid ruminal fraction, a total of 1706 bacterial ASVs agglomerated to 227 genera, 89 families, 51 orders, 27 classes and 16 phyla. Firmicutes (48.2%) Bacteroidetes (30.1%), Fibrobacterota (6.1%) were the three most abundant bacteria phyla (Fig. 1). *Prevotella_7* (9.7%), *Prevotella_1* (9.2%), unclassified Lachnospiraceae (7.6%), *Fibrobacter* (6.1%) and *Ruminococcus_1* (5.6%) were the 5 most dominant bacteria genera (Fig. 2). A total of 27 archaeal ASVs were identified and agglomerated to 4 genera (*Methanobrevibacter*, *Methanosphera*, *Methanimicrococcus* and *Candidatus methanomethylophilus*), three families, three orders three classes and one phylum. *Methanobrevibacter* was the most dominant archaeal genus (72.9%).

Alpha diversity analysis revealed that breed had an effect on solid associated bacterial and archaeal community richness and bacteria community PD (ANOVA, *P* < 0.05) (Table 2). Such differences were observed between the Cheviot and Lanark breeds, with the Cheviot exhibiting the least and the Lanark exhibiting the most rumen microbial diversity among the breeds. Based on weighted and unweighted UniFrac distances, beta diversity analysis showed no differences in overall community composition across the breeds (PERMANOVA, P > 0.05) for either bacterial or archaeal communities (Table 3).

**Table 2:** Alpha diversity analysis.

Bacteria community alpha diversity					
	Cheviot Mean ± Sd	Connemara Mean ± Sd	Lanark Mean ± Sd	Perth Mean ± Sd	Anova Pvalue
Solid fraction					
Shannon	4.1 ± 0.27	3.9 ± 0.24	4.2 ± 0.20	4.2 ± 0.27	0.202
InvSimpson	31.0 ± 8.75	20.0 ± 6.06	31.9 ± 7.02	32.8 ± 9.27	0.054
PD	48.3 ± 5.15^b^	54.3 ± 4.45^ab^	57.9 ± 6.97^a^	52.5 ± 7.10^ab^	0.036
Observed ASV	255.0 ± 41.5^b^	300.6 ± 33.73^ab^	334.9 ± 55.69^a^	291.6 ± 55.98^ab^	0.028
Liquid fraction					
Shannon	3.9 ± 0.27	3.9 ± 0.26	4.1 ± 0.21	3.9 ± 0.39	0.579
InvSimpson	23.2 ± 8.12	21.2 ± 7.71	28.1 ± 6.21	22.7 ± 13.05	0.464
PD	53.4 ± 7.68	52.6 ± 6.85	59.9 ± 6.81	52.4 ± 7.42	0.164
Observed ASV	284.4 ± 57.44	297.0 ± 49.15	338.7 ± 57.30	287.4 ± 51.21	0.157
Epithelial fraction					
Shannon	4.3 ± 0.19	4.1 ± 0.23	4.3 ± 0.28	4.1 ± 0.20	0.083
InvSimpson	33.5 ± 7.70^a^	17.7 ± 2.18^b^	34.9 ± 12.68^a^	19.6 ± 5.52^b^	0.001
PD	56.3 ± 3.53	65.8 ± 10.37	60.2 ± 6.19	63.0 ± 4.83	0.055
Observed ASV	313.7 ± 33.08^b^	417.7 ± 92.50^a^	340.0 ± 51.15^ab^	396.1 ± 48.58^ab^	0.032

Archaea community alpha diversity					
	Cheviot Mean ± Sd	Connemara Mean ± Sd	Lanark Mean ± Sd	Perth Mean ± Sd	Anova Pvalue
Solid fraction					
Shannon	1.1 ± 0.25	1.1 ± 0.41	1.3 ± 0.38	1.3 ± 0.25	0.389
InvSimpson	2.4 ± 0.52	2.5 ± 1.05	3.1 ± 0.98	3.0 ± 0.70	0.392
PD	1.6 ± 0.05	1.6 ± 0.10	1.7 ± 0.13	1.7 ± 0.15	0.129
Observed ASV	5.9 ± 1.13^b^	7.2 ± 1.10^ab^	9.3 ± 2.50^a^	8.9 ± 2.27^a^	0.001
Liquid fraction					
Shannon	1.1 ± 0.30	1.0 ± 0.38	1.2 ± 0.46	1.2 ± 0.36	0.636
InvSimpson	2.5 ± 0.74	2.1 ± 0.82	2.9 ± 1.19	3.1 ± 1.04	0.377
PD	1.6 ± 0.07	1.3 ± 0.55	1.7 ± 0.12	1.6 ± 0.07	0.085
Observed ASV	6.2 ± 1.39^b^	7.2 ± 2.49^ab^	9.4 ± 2.30^a^	7.0 ± 1.22^ab^	0.016
Epithelial fraction					
Shannon	1.2 ± 0.35	1.2 ± 0.29	1.3 ± 0.31	1.4 ± 0.30	0.785
InvSimpson	2.9 ± 1.27	2.3 ± 0.48	2.7 ± 0.99	3.2 ± 1.04	0.627
PD	1.6 ± 0.06	1.7 ± 0.14	1.7 ± 0.15	1.7 ± 0.18	0.157
Observed ASV	7.1 ± 2.47	9.3 ± 3.51	9.2 ± 1.83	9.4 ± 1.92	0.170

Measures of alpha diversity (Shannon, Simpson, Phylogenetic diversity and Observed ASV) for bacterial and archaeal communities, Mean ± Sd. Effect of breed on alpha diversity measures tested using two way ANOVA. Solid (Cheviot *n* = 8, Connemara *n* = 5, Lanark *n* = 9, Perth *n* = 7), liquid (Cheviot *n* = 9, Connemara *n* = 5, Lanark *n* = 9, Perth *n* = 5), epithelial (Cheviot *n* = 9, Connemara *n* = 3, Lanark *n* = 6, Perth *n* = 8).

**Table 3:** Beta diversity analysis.

Fraction	Weighted UniFrac		Unweighted UniFrac	
	PERMANOVA *P* value	R2	PERMANOVA *P* value	R2
Bacteria				
Solid	0.57	0.10	0.24	0.11
Liquid	0.59	0.10	0.27	0.12
Epithelial	0.47	0.12	0.09	0.14
Archaea				
Solid	0.72	0.08	0.45	0.11
Liquid	0.75	0.07	0.15	0.17
Epithelial	0.57	0.10	0.19	0.16

Effect of breed on bacterial and archaeal community composition in solid, liquid and epithelial ruminal fractions. Community dissimilarities calculated using weighted and unweighted UniFrac distances and compared among breeds using PERMANOVA, with P values and R2 values reported. Solid (Cheviot *n* = 8, Connemara *n* = 5, Lanark *n* = 9, Perth *n* = 7), liquid (Cheviot *n* = 9, Connemara *n* = 5, Lanark *n* = 9, Perth *n* = 5), epithelial (Cheviot *n* = 9, Connemara *n* = 3, Lanark *n* = 6, Perth *n* = 8).

The abundance of *Sharpea* at the genus level, *Sharpea azabuensis* at the species level and an unclassified ASV (ASV37) belonging to the family Lachnospiraceae were affected by breed (LRT, *P.adj* < 0.05) (Table 4). *Sharpea* (Wald, *P.*adj < 0.001; Log2FC = 4.37) and *Sharpea azabuensis* (Wald, *P.adj* < 0.001; Log2FC = 4.58) were higher in Perth compared to Cheviot. ASV37 (family Lachnospiraceae) was more abundant in Cheviot (Wald, *P.adj* < 0.01; Log2FC = 3.22) and Perth (Wald, *P.adj* < 0.001; Log2FC = 3.4) compared to Lanark (Table 4). Pairwise analysis between each of the breeds revealed a further 2 bacterial ASVs as differentially abundant. ASV48 classified to the genus *Prevotella_9* was higher in Lanark compared to Perth (Wald, *P.adj* < 0.0001; Log2FC = 7.88) and Cheviot (Wald, *P.adj* < 0.01; Log2FC = 9.11), and ASV329 classified to the genus *Pyramidobacter* was higher in Lanark compared to Cheviot (Wald, *P.adj* < 0.05; Log2FC = 5.52). At the genus level *P-2534-18B5_gut group* (ASV17), belonging to phylum Bacteroidetes, was higher in the Perth (Wald, *P.adj* < 0.05; Log2FC = 5.78) and Lanark (Wald, *P.adj* < 0.05; Log2FC = 6.82) breeds compared to Cheviot, and *Candidatus Saccharimonas* was higher in the Lanark compared to the Cheviot (Wald, *P.adj* < 0.05; Log2FC = 5.66). Similarly, at the family level P-2534-18B5_gut group (ASV17) was higher in the Perth (Wald, *P.adj* < 0.01; Log2FC = 5.88) and Lanark (Wald, *P.adj* < 0.01; Log2FC = 6.80) breeds compared to Cheviot, and Saccharimonadaceae (ASV317) was higher in the Lanark (Wald, *P.adj* < 0.01; Log2FC = 5.48) and Connemara (Wald, *P.adj* < 0.05; Log2FC = 6.89) breeds compared to the Cheviot. At the order level Coriobacteriales was higher in the Lanark compared to the Perth (Wald, *P.adj* < 0.05; Log2FC = 1.17) (Table 5). One archaea ASV belonging to the genus *Candidatus Methanomethylophilus* (ASV337) was higher in the Perth (Wald, *P.adj* < 0.01; Log2FC = 3.12) and Lanark (Wald, *P.adj* < 0.05; Log2FC = 3.21) compared to Cheviot (Table 5).

**Table 4:** Differential abundance analysis investigating the effect of breed on the abundance bacterial and archaeal taxa in solid, liquid and epithelial ruminal fractions.

	Rank	Classification	Cheviot Mean ± Sd	Connemara Mean ± Sd	Lanark Mean ± Sd	Perth Mean ± Sd	P.adj
Solid fraction							
ASV55	Genus	*Sharpea*	1.37 ± 0.35^b^	1.90 ± 0.71^ab^	1.55 ± 0.84^ab^	2.28 ± 0.51^a^	0.009
ASV55	ASV	*Sharpea azabuensis*	1.34 ± 0.34^b^	1.93 ± 0.73^ab^	1.58 ± 0.87^ab^	2.24 ± 0.51^a^	0.023
ASV37	ASV	F_Lachnospiraceae	2.22 ± 0.46^a^	2.40 ± 0.49^ab^	1.92 ± 0.50^b^	2.60 ± 0.53^a^	0.024
Epithelial fraction							
ASV247	Family	Family XIII	2.84 ± 0.22^b^	3.03 ± 0.17^ab^	3.33 ± 0.28^a^	2.95 ± 0.18^b^	< 0.001
ASV379	ASV	F_Family XIII	1.15 ± 0.39^ab^	1.44 ± 0.19^ab^	2.02 ± 0.47^a^	1.05 ± 0.43^b^	0.005

Analysis was conducted across all taxonomic ranks for bacterial populations (Phylum, Class, Order, Family, Genus and ASV), and lower taxonomic ranks for archaeal populations (Genus and ASV) using the LRT and Wald’s test from DESeq2. Table reports significant findings along with the log10 of normalised counts (Mean ± Sd), BH adjusted P values, pairwise comparisons (superscripts), taxonomic rank and classification. Solid (Cheviot *n* = 8, Connemara *n* = 5, Lanark *n* = 9, Perth *n* = 7), liquid (Cheviot *n* = 9, Connemara *n* = 5, Lanark *n* = 9, Perth *n* = 5), epithelial (Cheviot *n* = 9, Connemara *n* = 3, Lanark *n* = 6, Perth *n* = 8).

**Table 5:** Differential abundance analysis investigating the pairwise differences in bacterial and archaeal abundances between each of the breeds (i.e. Cheviot, Connemara, Lanark and Perth) in solid, liquid and epithelial ruminal fractions.

	Kingdom	Classification	Rank	Log2FC	P.adj	Comparison	Higher In
Solid							
ASV27	Bacteria	Coriobacteriales	Order	1.17	0.041	Perth v Lanark	Lanark
ASV17	Bacteria	p-2534-18B5_gut_group (Bacteroidetes)	Family	5.88	0.007	Perth v Cheviot	Perth
ASV17	Bacteria	p-2534-18B5_gut_group (Bacteroidetes)	Family	6.80	0.007	Lanark v Cheviot	Lanark
ASV317	Bacteria	Saccharimonadaceae	Family	5.48	0.007	Lanark v Cheviot	Lanark
ASV317	Bacteria	Saccharimonadaceae	Family	6.89	0.023	Conn. v Cheviot	Connemara
ASV17	Bacteria	F_p-2534-18B5_gut_group (Bacteroidetes)	Genus	5.78	0.012	Perth v Cheviot	Perth
ASV17	Bacteria	F_p-2534-18B5_gut_group (Bacteroidetes)	Genus	6.82	0.014	Lanark v Cheviot	Lanark
ASV55	Bacteria	*Sharpea*	Genus	4.37	0.000	Perth v Cheviot	Perth
ASV317	Bacteria	Candidatus_Saccharimonas	Genus	5.66	0.014	Lanark v Cheviot	Lanark
ASV37	Bacteria	F_Lachnospiraceae	ASV	3.22	0.010	Lanark v Cheviot	Cheviot
ASV48	Bacteria	G_*Prevotella 9*	ASV	9.11	0.010	Lanark v Cheviot	Lanark
ASV48	Bacteria	G_*Prevotella 9*	ASV	7.88	0.009	Perth v Lanark	Lanark
ASV37	Bacteria	F_Lachnospiraceae	ASV	3.38	0.000	Perth v Lanark	Perth
ASV55	Bacteria	*Sharpea azabuensis*	ASV	4.58	0.000	Perth v Cheviot	Perth
ASV329	Bacteria	G_*Pyramidobacter*	ASV	5.52	0.018	Lanark v Cheviot	Lanark
ASV337	Archaea	G_*Candidatus Methanomethylophilus*	ASV	3.21	0.023	Lanark v Cheviot	Lanark
ASV337	Archaea	G_*Candidatus Methanomethylophilus*	ASV	3.12	0.006	Perth v Cheviot	Perth
Liquid							
ASV2	Bacteria	Proteobacteria	Phylum	2.62	0.017	Perth v Conn	Perth
ASV223	Bacteria	Alphaproteobacteria	Class	7.11	0.002	Perth v Cheviot	Cheviot
ASV223	Bacteria	Alphaproteobacteria	Class	7.52	0.012	Conn. v Cheviot	Cheviot
ASV223	Bacteria	Rhodospirillales	Order	6.93	0.005	Perth v Cheviot	Cheviot
ASV223	Bacteria	Rhodospirillales	Order	7.29	0.024	Conn. v Cheviot	Cheviot
ASV219	Bacteria	Betaproteobacteriales	Order	3.03	0.045	Conn. v Cheviot	Cheviot
ASV223	Bacteria	O_Rhodospirillales	Family	7.11	0.021	Conn. v Cheviot	Cheviot
ASV223	Bacteria	O_Rhodospirillales	Family	6.81	0.003	Perth v Cheviot	Cheviot
ASV17	Bacteria	p-2534-18B5_gut_group (Bacteroidetes)	Family	4.75	0.047	Perth v Cheviot	Perth
ASV17	Bacteria	p-2534-18B5_gut_group (Bacteroidetes)	Family	5.87	0.019	Lanark v Cheviot	Lanark
ASV23	Bacteria	Muribaculaceae	Family	2.23	0.015	Perth v Cheviot	Cheviot
ASV461	Bacteria	O_Clostridiales *vadinBB60_group*	Family	5.82	0.049	Conn. v Cheviot	Cheviot
ASV461	Bacteria	O_Clostridiales *vadinBB60_group*	Family	6.09	0.004	Lanark v Conn	Lanark
ASV55	Bacteria	*Sharpea*	Genus	5.05	0.003	Perth v Cheviot	Perth
ASV223	Bacteria	O_Rhodospirillales	Genus	7.03	0.005	Perth v Cheviot	Cheviot
ASV461	Bacteria	O_Clostridiales *vadinBB60_group*	Genus	5.86	0.013	Lanark v Conn	Lanark
ASV20	Bacteria	F_Lachnospiraceae *NK3A20 group*	ASV	3.06	0.049	Lanark v Conn	Lanark
ASV23	Bacteria	F_Muribaculaceae	ASV	5.94	0.002	Perth v Cheviot	Cheviot
ASV43	Bacteria	F_Muribaculaceae	ASV	2.51	0.023	Perth v Cheviot	Cheviot
ASV44	Bacteria	G_*Acetitomaculum*	ASV	10.26	0.034	Conn. v Cheviot	Cheviot
ASV55	Bacteria	*Sharpea azabuensis*	ASV	4.81	0.006	Perth v Cheviot	Perth
Epithelial							
ASV38	Bacteria	Atopobiaceae	Family	1.96	0.014	Perth v Lanark	Lanark
ASV162	Bacteria	Synergistaceae	Family	2.19	0.018	Lanark v Cheviot	Lanark
ASV247	Bacteria	Family XIII	Family	1.41	0.000	Lanark v Cheviot	Lanark
ASV247	Bacteria	Family XIII	Family	1.13	0.008	Perth v Lanark	Lanark
ASV55	Bacteria	*Sharpea*	Genus	3.13	0.028	Perth v Cheviot	Perth
ASV69	Bacteria	F_Ruminococcaceae *UCG-014*	Genus	2.77	0.049	Lanark v Cheviot	Cheviot
ASV361	Bacteria	F_Family_XIII *AD3011_group*	Genus	2.28	0.006	Lanark v Cheviot	Lanark
ASV406	Bacteria	F_Family XIII *UCG-001*	Genus	3.10	0.049	Lanark v Cheviot	Cheviot
ASV24	Bacteria	G_*Succiniclasticum*	ASV	24.14	0.000	Conn. v Cheviot	Cheviot
ASV24	Bacteria	G_*Succiniclasticum*	ASV	23.67	0.000	Lanark v Conn	Lanark
ASV24	Bacteria	G_*Succiniclasticum*	ASV	25.98	0.000	Perth v Conn	Perth
ASV33	Bacteria	G_*Ruminococcus 1*	ASV	7.80	0.005	Conn. v Cheviot	Connemara
ASV33	Bacteria	G_*Ruminococcus 1*	ASV	4.81	0.023	Perth v Cheviot	Perth
ASV37	Bacteria	F_Lachnospiraceae	ASV	2.72	0.046	Perth v Lanark	Perth
ASV74	Bacteria	G_*Syntrophococcus*	ASV	5.42	0.049	Lanark v Conn	Lanark
ASV118	Bacteria	G_*Ruminococcus 1*	ASV	5.52	0.046	Perth v Lanark	Perth
ASV123	Bacteria	G_*Prevotella 1*	ASV	7.85	0.006	Lanark v Conn	Connemara
ASV379	Bacteria	F_Family_XIII	ASV	2.88	0.019	Perth v Lanark	Lanark
ASV633	Bacteria	F_Ruminococcaceae *UCG-010*	ASV	4.00	0.046	Perth v Lanark	Perth

Analysis was conducted across all taxonomic ranks for bacterial populations (Phylum, Class, Order, Family, Genus and ASV), and lower taxonomic ranks for archaeal populations (Genus and ASV) using the Wald’s pairwise test from DESeq2. Table reports the log10 of normalised counts (Mean ± Sd), BH adjusted P values, Log2 fold change, taxonomic rank and classification, breeds compared and the breed the abundance was increased in for significant findings. Solid (Cheviot *n* = 8, Connemara *n* = 5, Lanark *n* = 9, Perth *n* = 7), liquid (Cheviot *n* = 9, Connemara *n* = 5, Lanark *n* = 9, Perth *n* = 5), epithelial (Cheviot *n* = 9, Connemara *n* = 3, Lanark *n* = 6, Perth *n* = 8).

### Breed effects on bacterial and archaeal populations in the liquid ruminal fraction

For the liquid ruminal fraction, a total of 1790 bacteria ASVs agglomerated to 236 genera, 95 families, 57 orders, 29 classes and 17 phyla. Firmicutes (43.1%), Bacteroidetes (37.1%), and Proteobacteria (8.9%) were the most dominant phyla (Fig. 1). *Prevotella 7* (12.2%), *Prevotella 1* (11.7%), unclassified Lachnospiraceae (6.2%), *Succinivibrio* (5.8%) and *Succiniclasticum* (4.4%) were the five most dominant genera (Fig. 2). Twenty six archaea ASVs were available for analysis, which agglomerated to four genera (*Methanobrevibacter*, *Methanosphera,* unclassified Methanomethylophilaceae and *Candidatus Methanomethylophilus*), two families, two orders two classes and one phylum. *Methanobrevibacter* was the most dominant genus (78.9%).

Although there was no effect of breed on alpha diversity indices for bacteria communities (ANOVA, *P* > 0.05), breed did have an impact on the richness of archaeal communities. (ANOVA, *P* < 0.05) (Table 1). The Lanark breed had the highest level of archaeal community richness, whereas the Cheviot breed had the lowest level (Table 5). Based on weighted and unweighted UniFrac distances, the analysis of beta diversity showed no differences in overall community composition among breeds (PERMANOVA, *P* > 0.05) for either bacterial or archaeal communities (Table 3).

The likelihood ratio test detected no breed effect (LRT, *P.adj* > 0.05) on the abundance of bacterial or archaeal taxa across all taxonomic ranks. Pairwise analysis between each of the breeds revealed five taxa at the ASV level as differentially abundant. Two ASVs, ASV23 (Wald, *P.adj* < 0.01; Log2FC = 5.94) and ASV43 (Wald, *P.adj* < 0.05; Log2FC = 2.51) classified to the family Muribaculaceae were higher in Cheviot compared to Perth, ASV44 classified to the genus *Acetitomaculum* was higher in Cheviot (Wald, *P.adj* < 0.05; Log2FC = 10.26) compared to Connemara, ASV55 classified as *Sharpea azabuensis* was higher in Perth (Wald, *P.adj* < 0.01; Log2FC = 4.81) compared to Cheviot, and ASV20 classified to Lachnospiraceae *NK3A20 group* was higher in the Lanark (Wald, *P.adj* < 0.05; Log2FC = 3.06) compared to Connemara. At the genus level *Sharpea* (Wald, *P.adj* < 0.001; Log2FC = 5.05) was higher in the Perth compared to the Cheviot, ASV223 classified to order Rhodospirillales (Wald, *P.adj* < 0.01; Log2FC = 7.03) was higher in the Cheviot compared to the Perth, and ASV461 classified to Clostridiales_vadinBB60 group was higher in the Lanark (Wald, *P.adj* < 0.05; Log2FC = 5.86) compared to the Connemara. At the family level Muribaculaceae (Wald, *P.adj* < 0.05; Log2FC = 2.23) was higher in Cheviot compared to Perth, ASV223 classified to order Rhodospirillales was higher in the Cheviot compared to the Connemara (Wald, *P.adj* < 0.05; Log2FC = 7.11) and Perth (Wald, *P.adj* < 0.01; Log2FC = 6.81), ASV461 classified to Clostridiales_vadinBB60 group was higher in the Cheviot (Wald, *P.adj* < 0.05; Log2FC = 5.82) and Lanark (Wald, *P.adj* < 0.01; Log2FC = 6.09) compared to the Connemara, and ASV17 classified to P-2534-18B5_gut group was higher in the Perth (Wald, *P.adj* < 0.05; Log2FC = 4.75) and Lanark (Wald, *P.adj* < 0.05; Log2FC = 5.87) compared to the Cheviot. At the order level Rhodospirillales was higher in the Cheviot compared to the Connemara (Wald, *P.adj* < 0.05; Log2FC = 7.29) and Perth (Wald, *P.adj* < 0.01; Log2FC = 6.93), and Betaproteobacteriales was higher in the Cheviot compared to the Connemara (Wald, *P.adj* < 0.05; Log2FC = 3.03). At the class level the abundance of Alphaproteobacteria was higher in the Cheviot compared to the Perth (Wald, *P.adj* < 0.01; Log2FC = 7.11) and Connemara (Wald, *P.adj* < 0.05; Log2FC = 7.52). Finally at the phylum level the abundance of Proteobacteria was higher in the Perth compared to the Connemara (Wald, *P.adj* < 0.05; Log2FC = 2.62) (Table 5).

### Breed effects on bacterial and archaeal populations in the epithelial ruminal fraction

In the epithelial ruminal fraction, a total of 1891 bacteria ASVs agglomerated to 231 genera, 89 families, 52 orders, 29 classes and 17 phyla. Firmicutes (46.3%), Bacteroidetes (33.4%), and Proteobacteria (10.1%) were the most dominant phyla (Fig. 1). *Prevotella 1* (10.0%), *Prevotella 7* (8.9%), *Succinvibrio* (5.9%), unclassified Lachnospiraceae (5.7%), and *Ruminococcus 2* (5.0%) were the five most dominant genera (Fig. 2). Twenty eight archaeal ASVs were available for analysis, which agglomerated to five genera (*Methanobrevibacter*, *Methanosphera*, *Methanimicrococcus,* unclassified Methanomethylophilaceae and *Candidatus Methanomethylophilus*), three families, three orders, three classes and one phylum. *Methanobrevibacter* was the most dominant genus (82.0%).

Alpha diversity analysis revealed that while breed had no effect on epithelial associated archaeal community indices, it had a significant effect on bacteria community richness and inverse Simpson diversity (ANOVA, *P* < 0.05) (Table 2). Beta diversity analysis based on weighted and unweighted UniFrac distances, found no differences in community composition among the breeds (PERMANOVA, *P* > 0.05), for either bacterial or archaeal communities (Table 3).

The abundance of Family XIII at the family level and an unclassified ASV (ASV379) belonging to Family XIII at the ASV level were affected by breed (LRT, *P.adj* < 0.01) (Table 4). Family XIII was higher in Lanark compared to Cheviot (Wald, *P.adj* < 0.05; Log2FC = 1.41) and Perth (Wald, *P.adj* < 0.05; Log2FC = 1.13), and ASV379, belonging to Family XIII, was higher in the Lanark breed (Wald, *P.adj* < 0.05; Log2FC = 2.88) when compared to Perth breed (Table 5). Pairwise analysis revealed a further seven bacterial ASVs as differentially abundant. ASV37 classified to the Lachnospiraceae family was higher in the Perth (Wald, *P.adj* < 0.05; Log2FC = 2.72) compared to the Lanark, ASV123 classified to the genus *Prevotella_1* was higher in the Connemara (Wald, *P.adj* < 0.01; Log2FC = 7.85) compared to the Lanark, ASV633 classified to the genus Ruminococcacese *UCG*-*010* was higher in the Perth (Wald, *P.adj* < 0.05; Log2FC = 4.0) compared to the Lanark, ASV24 classified to the genus *Succiniclasticum* was lower in the Connemara compared to Cheviot (Wald, *P.adj* < 0.0001; Log2FC = 24.14), Lanark (Wald, P.adj < 0.0001; Log2FC = 23.67) and Perth (Wald, P.adj < 0.0001; Log2FC = 25.98) breeds, ASV74 classified to the genus *Syntrophococcus* was higher in the Lanark breed compared to Connemara (Wald, *P.adj* < 0.05; Log2FC = 5.42), ASV33 classified to the genus *Ruminococcus_1* was higher in Perth (Wald, *P.adj* < 0.05; Log2FC = 4.81) and Connemara (Wald, *P.adj* < 0.01; Log2FC = 7.80) compared to Cheviot, and ASV118 also classified to the genus *Ruminococcus_1* was higher in Perth (Wald, *P.adj* < 0.05; Log2FC = 5.52) compared to Lanark. At the genus level, *Sharpea* was higher in the Perth compared to the Cheviot (Wald, *P.adj* < 0.05; Log2FC = 3.13), ASV361 classified to Family XIII *AD3011 group* was higher in the Lanark (Wald, *P.adj* < 0.01; Log2FC = 2.28) when compared to the Cheviot, while ASV69 classified to Ruminococcaceae *UCG-014* (Wald, *P.adj* < 0.05; Log2FC = 2.77) and ASV406 classified to Family XIII *UCG-001* (Wald, *P.adj* < 0.05; Log2FC = 3.10) were both higher in the Cheviot when compared to the Lanark. At the family level Atopobiaceae was higher in the Lanark (Wald, *P.adj* < 0.05; Log2FC = 1.96) compared to Perth, and Synergistaceae was higher in the Lanark compared to the Cheviot (Wald, *P.adj* < 0.05; Log2FC = 2.19) (Table 5).

### Effect of ruminal fraction on bacterial and archaeal populations across breeds

**Figure 3:**
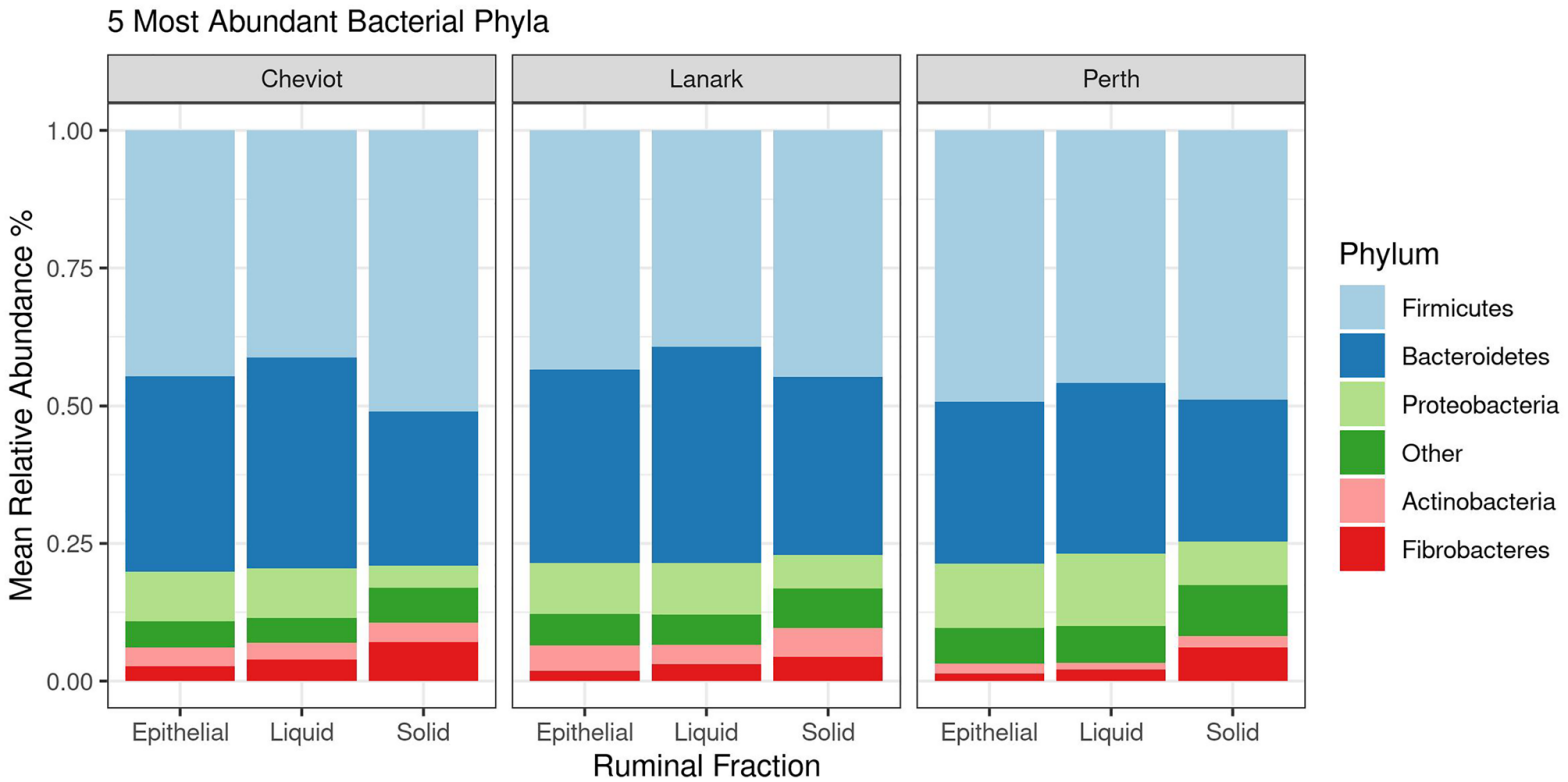
Stack barchart representing the mean relative abundance of the 5 most dominant phyla across ruminal fractions (i.e. solid, liquid and epithelial) for Cheviot, Lanark and Perth breeds. Cheviot (solid *n* = 7, liquid *n* = 5, epithelial *n* = 5), Lanark (solid *n* = 7, liquid *n* = 5, epithelial *n* = 5), Perth (solid *n* = 7, liquid *n* = 5, epithelial *n* = 5).

**Figure 4:**
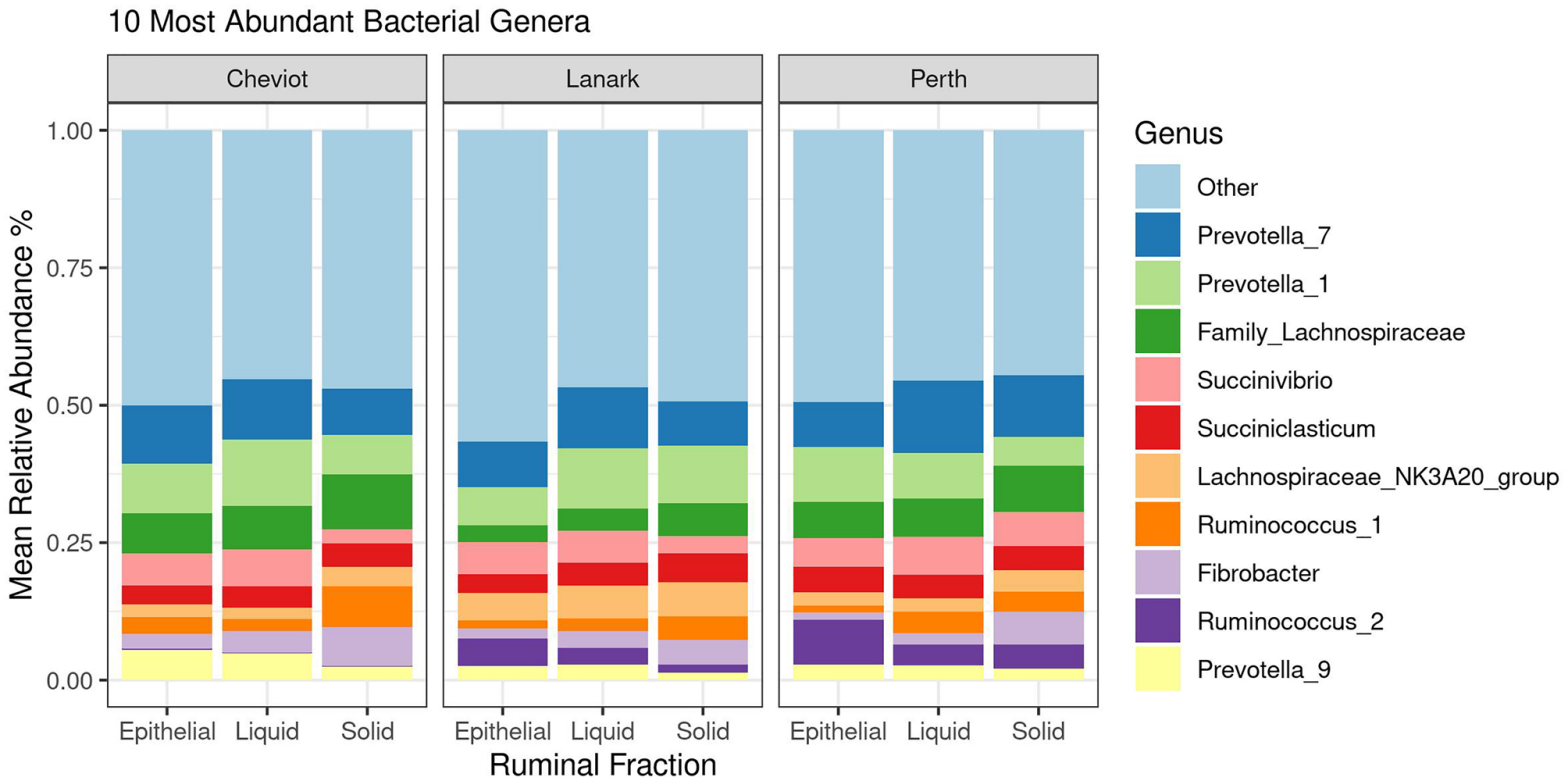
Stack barchart representing the mean relative abundance of the 10 most dominant bacterial genera across ruminal fractions (i.e. solid, liquid and epithelial) for Cheviot, Lanark and Perth breeds. Cheviot (solid *n* = 7, liquid *n* = 5, epithelial *n* = 5), Lanark (solid *n* = 7, liquid *n* = 5, epithelial *n* = 5), Perth (solid *n* = 7, liquid *n* = 5, epithelial *n* = 5).

Bacterial and archaeal populations across ruminal fractions were investigated for Cheviot, Lanark and Perth breeds, and only included animals where all three ruminal fractions were available. Firmicutes was the most abundant phylum in the Cheviot (mean, solid = 51%, liquid = 41%, epithelial = 45%), Lanark (mean, solid = 44%, liquid = 39%, epithelial = 43%) and Perth (mean, solid = 49%, liquid = 46%, epithelial = 49%) breeds (Fig. 3). In the epithelial fraction *Prevotella*_7 was the most abundant genus in the Cheviot (10.6%) and Lanark (8.3%) breeds, while *Prevotella_1* (10.0%) was the most dominant genus for the Perth breed. In the liquid fraction *Prevotella_1* was the most dominant in the Cheviot (12.1%) breed, while *Prevotella_7* was most dominant in Lanark (11.1%) and Perth (13.1%) breeds. In the solid ruminal fraction unclassified Lachnospiracheae, *Prevotella_1* and *Prevotella_7* were most abundant in the Cheviot (9.9%), Lanark (10.5%) and Perth (11.1%) breeds, respectively (Fig. 4).

For the Cheviot breed, bacterial community alpha diversity measures were not affected by ruminal fraction (ANOVA, *P* > 0.1). For the Lanark breed, bacterial community Shannon diversity was affected by ruminal fraction (ANOVA, *P* < 0.05). For the Perth breed, bacterial community richness (observed ASV) and phylogenetic diversity (PD) were affected by ruminal fraction (ANOVA, *P* < 0.05), with the rumen epithelial fraction exhibiting greater diversity than solid and liquid ruminal fractions (Table 6). For all three breeds, archaeal community alpha diversity measures were not affected by ruminal fraction (ANOVA, *P* > 0.1). Beta diversity analysis showed that bacterial and archaeal community composition were also unaffected by ruminal fraction for all breeds analysed (PERMANOVA, *P* > 0.1) (Table 7).

**Table 6:** Alpha diversity analysis.

Bacteria community alpha diversity				
	Epithelial Mean ± Sd	Liquid Mean ± Sd	Solid Mean ± Sd	Anova Pvalue
Cheviot				
Shannon	4.2 ± 0.20	4.0 ± 0.28	4.1 ± 0.18	0.118
InvSimpson	31.7 ± 6.86	25.0 ± 8.41	33.1 ± 6.78	0.121
PD	55.7 ± 3.01	53.8 ± 8.53	48.7 ± 5.43	0.112
Observed ASV	305.4 ± 32.99	288.1 ± 65.09	257.4 ± 44.20	0.21
Lanark				
Shannon	4.4 ± 0.16^a^	4.2 ± 0.16^b^	4.4 ± 0.13^ab^	0.037
InvSimpson	38.2 ± 10.95	29.5 ± 5.97	35.7 ± 4.04	0.174
PD	61.1 ± 6.54	59.6 ± 4.82	59.6 ± 7.59	0.908
Observed ASV	344.8 ± 55.66	341.0 ± 34.40	354.0 ± 54.83	0.904
Perth				
Shannon	4.1 ± 0.14	3.9 ± 0.39	4.2 ± 0.31	0.476
InvSimpson	21.2 ± 5.38	22.7 ± 13.05	32.0 ± 10.69	0.264
PD	62.1 ± 5.63^a^	52.4 ± 7.42^ab^	51.5 ± 8.35^b^	0.03
Observed ASV	362.8 ± 61.58^a^	287.4 ± 51.21^b^	285.8 ± 65.14^b^	0.027

Archaea community alpha diversity				
	Epithelial Mean ± Sd	Liquid Mean ± Sd	Solid Mean ± Sd	Anova Pvalue
Cheviot				
Shannon	1.2 ± 0.24	1.1 ± 0.30	1.1 ± 0.25	0.752
InvSimpson	2.6 ± 0.76	2.5 ± 0.75	2.4 ± 0.53	0.800
PD	1.6 ± 0.06	1.6 ± 0.07	1.5 ± 0.04	0.122
Observed ASV	6.4 ± 1.27	6.6 ± 1.27	5.9 ± 1.07	0.517
Lanark				
Shannon	1.3 ± 0.31	1.2 ± 0.46	1.3 ± 0.38	0.900
InvSimpson	2.7 ± 0.99	2.9 ± 1.19	3.1 ± 0.98	0.847
PD	1.7 ± 0.15	1.7 ± 0.12	1.7 ± 0.13	0.737
Observed ASV	9.2 ± 1.83	9.9 ± 2.37	9.3 ± 50	0.818
Perth				
Shannon	1.5 ± 0.05	1.2 ± 0.36	1.3 ± 0.30	0.320
InvSimpson	3.6 ± 0.50	3.1 ± 1.04	3.1 ± 0.84	0.565
PD	1.8 ± 0.20	1.6 ± 0.07	1.7 ± 0.16	0.534
Observed ASV	8.8 ± 2.17	7.0 ± 1.22	8.2 ± 2.17	0.293

Measures of alpha diversity (Shannon, Inverse Simpson, Phylogenetic diversity and Observed ASV) for bacterial and archaeal communities, reported as Mean ± Sd. Effect of breed on alpha diversity tested using 2 way ANOVA. Cheviot (solid *n* = 7, liquid *n* = 7, epithelial *n* = 7), Lanark (solid *n* = 5, liquid *n* = 5, epithelial *n* = 5), Perth (solid *n* = 5, liquid *n* = 5, epithelial *n* = 5).

**Table 7:** Beta diversity analysis.

Breed	Weighted UniFrac		Unweighted UniFrac	
	PERMANOVA *P* value	R2	PERMANOVA *P* value	R2
Bacteria				
Cheviot	0.49	0.09	0.89	0.06
Perth	0.68	0.09	0.64	0.11
Lanark	0.8	0.1	0.85	0.08
Archaea				
Cheviot	0.95	0.02	0.89	0.03
Perth	0.3	0.17	0.82	0.06
Lanark	0.6	0.9	0.83	0.06

Effect of fraction on bacterial and archaeal community composition for Cheviot, Lanark and Perth breeds. Community dissimilarities calculated using weighted and unweighted UniFrac distances and compared among breeds using PERMANOVA. *P* values and R2 values reported. Cheviot (solid *n* = 7, liquid *n* = 7, epithelial *n* = 7), Lanark (solid *n* = 5, liquid *n* = 5, epithelial *n* = 5), Perth (solid *n* = 5, liquid *n* = 5, epithelial *n* = 5).

Overall, ruminal fraction influenced 36 taxonomic groups across all ranks, representing 19 distinct ASVs, in the three breeds studied (LRT, *P* < 0.05). Ruminal fraction influenced the abundance of 18 taxa (11 distinct ASVs) in the Lanark breed, the most of any of breeds studied. ASV141, classified to the phylum Epsilonbacteraeota and the genus *Campylobacter,* was affected by ruminal fraction (LRT, *P* < 0.05) at all taxonomic ranks (i.e. phylum to ASV) and found to be significantly more abundant in the epithelial fraction when compared to the solid fraction. The abundance of ASV449, classified to the genus *Desulfobulbus,* was affected by ruminal fraction at the order, family and genus taxonomic ranks (LRT, *P.adj* < 0.05), found to be significantly more abundan in the epithelial ruminal fraction. At the genus level the abundance of *Butyrivibrio 2*, *Fretibacterium*, *Howardella,* and an unclassified ASV (ASV219) belonging to family Neisseriaceae were all affected by ruminal fraction (LRT, *P* < 0.05) and significantly higher in the epithelial fraction. Conversely, the abundance of *Shutterella* and two unclassified ASVs belonging to families Family XII *UCG-001* and Eggerthellaceae were highest in the solid ruminal fraction (Wald, *P.adj* < 0.05). At the ASV level, the abundance of two unclassified ASVs; ASV210 and ASV239, belonging to genus *Mogibacterium* and family Family XIII were affected by ruminal fraction (LRT, *P* < 0.05) and highest in the epithelial ruminal fraction (Wald, *P* < 0.05). In the Cheviot breed, the abundance of 15 taxa (8 unique ASVs) were affected by ruminal fraction. ASV141 (*Campylobacter*) from taxonomic ranks phylum to genus and ASV449 (*Desulfobulbus*) from order to genus were differentially abundant and significantly more abundant in the epithelial ruminal fraction (Wald, *P* < 0*.*05). At the family level, Neisseriaceae and an unclassified ASV, ASV198, belonging to the order Coriobacteriales, were affected by ruminal fraction, with the epithelial and solid ruminal fractions, respectively, containing a higher proportion of these bacteria. At the genus level, the abundance of *Mogibacterium*, *Butyrivibro 2* and two unclassified ASVs, ASV142 (F_Erysipelotrichaceae_UCG-004) and ASV263 (F_Burkholderiaceae), were affected by ruminal fraction (LRT, *P* < 0.05), with highest abundances observed in the epithelial ruminal fraction. In the Perth breed, the abundance of the bacterial phylum Tenericutes and an unclassified archaeal genus, ASV475, belonging to the family Methanomethylophilaceae were impacted by ruminal fraction (LRT, P0.05) (Table 8) Taken together, the majority of differences in microbial abundance were observed between the solid and epithelial ruminal fractions, as shown in Table 9, which summarises all the results of pairwise analysis between fractions.

**Table 8:** Differential abundance analysis investigating the effect of ruminal fraction on the abundance bacterial and archaeal taxa in Cheviot, Lanark and Perth breeds.

	Kingdom	Classification	Rank	P.adj	Epithelial	Liquid	Solid
Cheviot							
ASV141	Bacteria	Epsilonbacteraeota	Phylum	0.003	1.72 ± 0.89^a^	1.14 ± 0.71^ab^	0.68 ± 0.48^b^
ASV141	Bacteria	Campylobacteria	Class	0.002	1.72 ± 0.89^a^	1.14 ± 0.71^ab^	0.65 ± 0.48^b^
ASV141	Bacteria	Campylobacterales	Order	0.005	1.71 ± 0.92^a^	1.11 ± 0.70^ab^	0.67 ± 0.50^b^
ASV219	Bacteria	Betaproteobacteriales	Order	0.028	2.32 ± 0.52^a^	1.98 ± 0.44^ab^	1.80 ± 0.24^b^
ASV449	Bacteria	Desulfobacterales	Order	0.028	1.79 ± 0.45^a^	1.19 ± 0.71^ab^	0.84 ± 0.45^b^
ASV141	Bacteria	Campylobacteraceae	Family	0.002	1.72 ± 0.89^a^	1.12 ± 0.71^ab^	0.64 ± 0.48^b^
ASV198	Bacteria	O_Coriobacteriales	Family	0.000	1.50 ± 0.26^b^	1.56 ± 0.17^b^	2.03 ± 0.13^a^
ASV219	Bacteria	Neisseriaceae	Family	0.000	1.84 ± 0.60^a^	1.42 ± 0.58^b^	1.00 ± 0.53^b^
ASV449	Bacteria	Desulfobulbaceae	Family	0.014	1.81 ± 0.49^a^	1.19 ± 0.73^ab^	0.81 ± 0.44^b^
ASV141	Bacteria	*Campylobacter*	Genus	0.011	1.74 ± 0.97^a^	1.15 ± 0.73^ab^	0.64 ± 0.50^b^
ASV142	Bacteria	F_Erysipelotrichaceae *UCG-004*	Genus	0.030	1.65 ± 0.75^a^	1.88 ± 0.86^b^	1.84 ± 0.47^ab^
ASV199	Bacteria	*Mogibacterium*	Genus	0.030	1.69 ± 0.55^a^	1.38 ± 0.52^ab^	0.94 ± 0.92^b^
ASV254	Bacteria	*Butyrivibrio 2*	Genus	0.022	2.22 ± 0.62^a^	1.43 ± 0.78^ab^	0.96 ± 0.63^b^
ASV263	Bacteria	F_Burkholderiaceae	Genus	0.022	1.52 ± 0.58^a^	1.00 ± 0.64^ab^	0.55 ± 0.42^b^
ASV449	Bacteria	*Desulfobulbus*	Genus	0.030	1.82 ± 0.40^a^	1.22 ± 0.75^ab^	0.80 ± 0.43^b^
Lanark							
ASV141	Bacteria	Epsilonbacteraeota	Phylum	0.002	2.94 ± 0.54^a^	1.03 ± 0.72^b^	0.57 ± 0.61^b^
ASV141	Bacteria	Campylobacteria	Class	0.009	2.36 ± 0.54^a^	1.03 ± 0.72^ab^	0.57 ± 0.61^b^
ASV141	Bacteria	Campylobacterales	Order	0.003	2.34 ± 0.58^a^	1.01 ± 0.66^b^	0.58 ± 0.62^b^
ASV421	Bacteria	Desulfobacterales	Order	0.000	2.26 ± 0.45^a^	0.84 ± 0.61^b^	0.64 ± 0.42^b^
ASV141	Bacteria	Campylobacteraceae	Family	0.003	2.33 ± 0.58^a^	1.04 ± 0.69^b^	0.59 ± 0.63^b^
ASV421	Bacteria	Desulfobulbaceae	Family	0.000	2.25 ± 0.42^a^	0.85 ± 0.61^b^	0.62 ± 0.39^b^
ASV141	Bacteria	*Campylobacter*	Genus	0.001	2.41 ± 0.62^a^	1.02 ± 0.67^b^	0.53 ± 0.56^b^
ASV219	Bacteria	F_Neisseriaceae	Genus	0.013	1.70 ± 0.64^a^	0.87 ± 0.35^ab^	0.37 ± 0.51^b^
ASV239	Bacteria	*Butyrivibrio 2*	Genus	0.008	2.05 ± 1.30^a^	0.79 ± 0.74^b^	0.48 ± 0.50^b^
ASV391	Bacteria	*Fretibacterium*	Genus	0.000	2.02 ± 1.16^a^	1.00 ± 0.56^b^	0.40 ± 0.39^b^
ASV406	Bacteria	Family XIII *UCG-001*	Genus	0.000	0.55 ± 0.54^b^	0.90 ± 0.59^ab^	1.43 ± 0.41^a^
ASV421	Bacteria	*Desulfobulbus*	Genus	0.000	2.33 ± 0.51^a^	0.85 ± 0.62^b^	0.59 ± 0.38^b^
ASV523	Bacteria	F_Eggerthellaceae	Genus	0.012	1.33 ± 0.20^b^	1.39 ± 0.15^ab^	1.75 ± 0.11^a^
ASV587	Bacteria	*Shuttleworthia*	Genus	0.017	0.69 ± 0.46^b^	1.03 ± 0.43^ab^	1.47 ± 0.14^a^
ASV846	Bacteria	*Howardella*	Genus	0.018	1.68 ± 0.13^a^	0.74 ± 0.70^ab^	0.87 ± 0.24^b^
ASV141	Bacteria	*Campylobacter*	ASV	0.002	2.43 ± 0.63^a^	1.01 ± 0.65^b^	0.50 ± 0.54^b^
ASV210	Bacteria	*Mogibacterium*	ASV	0.024	2.28 ± 0.37^a^	1.59 ± 0.40^ab^	0.97 ± 0.63^b^
ASV379	Bacteria	F_Family XIII	ASV	0.000	2.06 ± 0.44^a^	0.82 ± 0.53^b^	0.20 ± 0.29^b^
Perth							
ASV126	Bacteria	Tenericutes	Phylum	0.015	2.33 ± 0.25^b^	2.6 ± 0.26^a^	2.55 ± 0.19^ab^
ASV475	Archaea	F_Methanomethylophilaceae	Genus	0.019	1.43 ± 1.03^a^	0.52 ± 0.48^b^	0.42 ± 0.45^b^

Analysis was conducted across all taxonomic ranks for bacterial populations (Phylum, Class, Order, Family, Genus and ASV), and lower taxonomic ranks for archaeal populations (Genus and ASV) using the LRT from DESeq2. Table reports significant findings along with the log10 of normalised counts (Mean ± Sd), BH adjusted P values, pairwise comparisons (superscripts). Taxonomic rank and classification. Cheviot (solid *n* = 7, liquid *n* = 7, epithelial *n* = 7), Lanark (solid *n* = 5, liquid *n* = 5, epithelial *n* = 5), Perth (solid *n* = 5, liquid *n* = 5, epithelial *n* = 5).

**Table 9:** Differential abundance analysis investigating the pairwise differences in bacterial and archaeal abundances between each of the fractions (i.e. solid, liquid and epithelial) in the Cheviot, Lanark and Perth breeds.

	Kingdom	Classification	Rank	Log2FC	P.adj	Comparison	Higher in
Cheviot							
ASV141	Bacteria	Epsilonbacteraeota	Phylum	5.42	0.000	Solid v epithelial	Epithelial
ASV3	Bacteria	Firmicutes	Phylum	0.63	0.023	Solid v epithelial	Solid
ASV14	Bacteria	Fibrobacter	Phylum	1.95	0.023	Solid v epithelial	Solid
ASV81	Bacteria	Spirochaetes	Phylum	1.97	0.050	Solid v epithelial	Solid
ASV141	Bacteria	Campylobacteria	Class	5.51	0.000	Solid v epithelial	Epithelial
ASV141	Bacteria	Campylobacterales	Order	5.26	0.000	Solid v epithelial	Epithelial
ASV449	Bacteria	Desulfobacterales	Order	3.48	0.002	Solid v epithelial	Epithelial
ASV219	Bacteria	Betaproteobacteriales	Order	2.53	0.005	Solid v epithelial	Epithelial
ASV219	Bacteria	Neisseriaceae	Family	3.31	0.002	Liquid v epithelial	Epithelial
ASV141	Bacteria	Campylobacteraceae	Family	5.15	0.000	Solid v epithelial	Epithelial
ASV449	Bacteria	Desulfobulbaceae	Family	3.74	0.001	Solid v epithelial	Epithelial
ASV219	Bacteria	Neisseriaceae	Family	3.51	0.000	Solid v epithelial	Epithelial
ASV3	Bacteria	Acidaminococcaceae	Family	0.98	0.042	Solid v epithelial	Solid
ASV198	Bacteria	O_Coriobacteriales	Family	1.59	0.001	Solid v epithelial	Solid
ASV81	Bacteria	Spirochaetaceae	Family	2.12	0.042	Solid v epithelial	Solid
ASV198	Bacteria	O_Coriobacteriales	Family	1.57	0.005	Solid v liquid	Solid
ASV142	Bacteria	F_Erysipelotrichaceae *UCG-004*	Genus	3.50	0.025	Liquid v epithelial	Liq
ASV141	Bacteria	Campylobacter	Genus	5.70	0.000	Solid v epithelial	Epithelial
ASV254	Bacteria	Butyrivibrio_2	Genus	4.16	0.001	Solid v epithelial	Epithelial
ASV263	Bacteria	F_Burkholderiaceae	Genus	4.02	0.001	Solid v epithelial	Epithelial
ASV128	Bacteria	F_Lachnospiraceae *UCG-008*	Genus	3.73	0.018	Solid v epithelial	Epithelial
ASV199	Bacteria	Mogibacterium	Genus	3.70	0.002	Solid v epithelial	Epithelial
ASV449	Bacteria	Desulfobulbus	Genus	3.63	0.002	Solid v epithelial	Epithelial
ASV347	Bacteria	Family XIII *AD3011 group*	Genus	2.56	0.032	Solid v epithelial	Epithelial
ASV141	Bacteria	G_Campylobacter	ASV	6.15	0.001	Solid v epithelial	Epithelial
ASV263	Bacteria	F_Burkholderiaceae	ASV	4.32	0.006	Solid v epithelial	Epithelial
Lanark							
ASV141	Bacteria	Epsilonbacteraeota	Phylum	4.09	0.023	Liquid v epithelial	Epithelial
ASV141	Bacteria	Epsilonbacteraeota	Phylum	5.88	0.000	Solid v epithelial	Epithelial
ASV41	Bacteria	Fibrobacter	Phylum	1.34	0.035	Solid v epithelial	Solid
ASV141	Bacteria	Campylobacteria	Class	5.68	0.001	Solid v epithelial	Epithelial
ASV421	Bacteria	Desulfobacterales	Order	4.57	0.001	Liquid v epithelial	Epithelial
ASV141	Bacteria	Campylobacterales	Order	4.39	0.011	Liquid v epithelial	Epithelial
ASV1	Bacteria	Bacteroidales	Order	0.63	0.021	liquid v epithelial	Liquid
ASV141	Bacteria	Campylobacterales	Order	5.76	0.000	Solid v epithelial	Epithelial
ASV421	Bacteria	Desulfobacterales	Order	5.63	0.000	Solid v epithelial	Epithelial
ASV41	Bacteria	Fibrobacterales	Order	1.55	0.011	Solid v epithelial	Solid
ASV421	Bacteria	Desulfobulbaceae	Family	4.45	0.001	Liquid v epithelial	Epithelial
ASV141	Bacteria	Campylobacteraceae	Family	4.20	0.024	Liquid v epithelial	Epithelial
ASV141	Bacteria	Campylobacteraceae	Family	5.77	0.000	Solid v epithelial	Epithelial
ASV421	Bacteria	Desulfobulbaceae	Family	5.77	0.000	Solid v epithelial	Epithelial
ASV219	Bacteria	Neisseriaceae	Family	4.38	0.005	Solid v epithelial	Epithelial
ASV41	Bacteria	Fibrobacteraceae	Family	1.49	0.019	Solid v epithelial	Solid
ASV239	Bacteria	Butyrivibrio 2	Genus	5.41	0.031	Liquid v epithelial	Epithelial
ASV421	Bacteria	Desulfobulbus	Genus	4.95	0.002	Liquid v epithelial	Epithelial
ASV391	Bacteria	Fretibacterium	Genus	4.78	0.014	Liquid v epithelial	Epithelial
ASV141	Bacteria	Campylobacter	Genus	4.73	0.014	Liquid v epithelial	Epithelial
ASV391	Bacteria	Fretibacterium	Genus	7.48	0.000	Solid v epithelial	Epithelial
ASV239	Bacteria	Butyrivibrio 2	Genus	7.07	0.001	Solid v epithelial	Epithelial
ASV141	Bacteria	Campylobacter	Genus	6.56	0.000	Solid v epithelial	Epithelial
ASV421	Bacteria	Desulfobulbus	Genus	6.37	0.000	Solid v epithelial	Epithelial
ASV219	Bacteria	F_Neisseriaceae	Genus	5.21	0.001	Solid v epithelial	Epithelial
ASV708	Bacteria	Bacteroides	Genus	3.70	0.020	Solid v epithelial	Epithelial
ASV568	Bacteria	Alistipes	Genus	3.39	0.016	Solid v epithelial	Epithelial
ASV846	Bacteria	Howardella	Genus	2.78	0.011	Solid v epithelial	Epithelial
ASV523	Bacteria	F_Eggerthellaceae	Genus	1.39	0.011	Solid v epithelial	Solid
ASV587	Bacteria	Shuttleworthia	Genus	2.49	0.005	Solid v epithelial	Solid
ASV406	Bacteria	Family XIII *UCG-001*	Genus	3.19	0.000	Solid v epithelial	Solid
ASV141	Bacteria	G_Campylobacter	ASV	5.02	0.017	Liquid v epithelial	Epithelial
ASV379	Bacteria	F_Family XIII	ASV	4.40	0.014	Liquid v epithelial	Epithelial
ASV379	Bacteria	F_Family XIII	ASV	7.53	0.000	Solid v epithelial	Epithelial
ASV141	Bacteria	G_Campylobacter	ASV	6.93	0.000	Solid v epithelial	Epithelial
ASV247	Bacteria	F_Family XIII	ASV	5.87	0.005	Solid v epithelial	Epithelial
ASV361	Bacteria	F_Family XIII *AD3011 group*	ASV	5.72	0.004	Solid v epithelial	Epithelial
ASV210	Bacteria	G_Mogibacterium	ASV	4.09	0.002	Solid v epithelial	Epithelial
Perth							
ASV126	Bacteria	Tenericutes	Phylum	0.97	0.003	Liquid v epithelial	Liquid
ASV421	Bacteria	Desulfobacterales	Order	3.73	0.007	Solid v epithelial	Epithelial
ASV102	Bacteria	Family XIII	Family	1.27	0.050	Solid v liquid	Solid
ASV219	Bacteria	Neisseriaceae	Family	4.24	0.027	Solid v epithelial	Epithelial
ASV391	Bacteria	Fretibacterium	Genus	6.89	0.006	Solid v epithelial	Epithelial
ASV421	Bacteria	Desulfobulbus	Genus	4.25	0.006	Solid v epithelial	Epithelial
ASV276	Bacteria	O_Clostridiales	ASV	6.27	0.050	Solid v epithelial	Epithelial
ASV128	Bacteria	F_Lachnospiraceae *UCG-008*	ASV	4.63	0.036	Solid v epithelial	Epithelial

Analysis was conducted across all taxonomic ranks for bacterial populations (Phylum, Class, Order, Family, Genus and ASV), and lower taxonomic ranks for archaeal populations (Genus and ASV) using the Wald’s pairwise test from DESeq2. Table reports significant findings along with the log10 of normalised counts (Mean ± Sd), BH adjusted P values, Log2 fold change, taxonomic rank and classification, fractions compared, and the fraction the abundance was increased in. Cheviot (solid *n* = 7, liquid *n* = 7, epithelial *n* = 7), Lanark (solid *n* = 5, liquid *n* = 5, epithelial *n* = 5), Perth (solid *n* = 5, liquid *n* = 5, epithelial *n* = 5).

### Bacterial and archaeal genera associated with FCR and ADG

Spearman's correlation analysis was performed between the relative abundance of genera and animal production traits; FCR and ADG to find potential drivers of feed efficiency in the solid, liquid and epithelial fractions. After adjusting for repeated hypotheses testing, no genera were determined to be statistically significant. Therefore, putative drivers of FCR and ADG were considered to have a (P < 0.05). In the solid fraction, four bacterial genera showed significant negative correlations with FCR: *Succinivibrionaceae* (ρ = − 4.1), *Lachnospira* (ρ = − 3.9), *Syntrophococcus* (ρ = − 3.8) and an unclassified genus (ASV9) belonging to the order Gastranaerophilales (ρ = − 4.1). Ruminococcaceae *UCG-013* (ρ = − 4.1) positively associated with ADG, while Lachnospiraceae *NK3A20 group* (ρ = − 3.8) was negatively correlated with ADG. In the liquid ruminal fraction the genus *Acetitomaculum* (ρ = − 3.8) an unclassified ASV belonging to the order Gastranaerophilales (ρ = − 4.4) and the archaeal genus *Candidatus Methanomethylophilus* (ρ = − 3.8) negatively correlated with FCR. Prevotella 9 (ρ = 3.8), Roseburia (ρ = 4.9), and 5 unclassified genera belonging to the families Ruminococcaceae-*UCG-013* (ρ = 4.5), -*UCG-002* (ρ = 4.4), -*UCG-014* (ρ = 4.1), -*UCG-010* (ρ = 4.1), and Lachnospiraceae (ρ = 3.9), and an unclassified genus belonging to order Mollicutes (ρ = 4.9) positively associated with ADG. In the epithelial fraction we observed no significant associations with FCR. *Prevotella 9* (ρ = 4.8), 4 unclassified genera belonging to the families Ruminococcaceae-*UCG-013* (ρ = 4.9), -*UCG-009* (ρ = 4.7), -*UCG-014* (ρ = 4.1) and Lachnospiraceae (ρ = 4.5), an unclassified genus belonging to order Mollicutes_RF39 (ρ = 4.6) and the archaeal genus *Methanosphera* (ρ = 4.6) positively associated with ADG. *Mogibacterium* (ρ = − 4.1) and 2 unclassified genera belonging to the families Prevotellaceae (ρ = − 4.3) and Christensenellaceae (ρ = 4.3) negatively associated with ADG (Table 10).

**Table 10:** Spearman’s rank correlation of bacterial and archaeal genera in the solid, liquid, and epithelial ruminal fractions that had a significant association with animal production traits FCR and/or ADG.

	Kingdom	Classification	ρ	P value	P.adj	Trait
Solid						
ASV21	Bacteria	Succinivibrionaceae	− 0.41	0.025	0.707	FCR
ASV71	Bacteria	*Syntrophococcus*	− 0.38	0.037	0.707	FCR
ASV149	Bacteria	*Lachnospira*	− 0.39	0.033	0.707	FCR
ASV9	Bacteria	O_Gastranaerophilales	− 0.41	0.026	0.707	FCR
ASV16	Bacteria	F_Lachnospiraceae *NK3A20 group*	− 0.38	0.038	0.913	ADG
ASV366	Bacteria	F_Ruminococcaceae *UCG-013*	0.38	0.040	0.913	ADG
Liquid						
ASV9	Bacteria	O_Gastranaerophilales	− 0.44	0.018	0.904	FCR
ASV44	Bacteria	*Acetitomaculum*	− 0.38	0.043	0.904	FCR
ASV337	Archaea	Candidatus_Methanomethylophilus	− 0.38	0.044	0.177	FCR
ASV10	Bacteria	F_Lachnospiraceae	0.39	0.038	0.434	ADG
ASV32	Bacteria	*Prevotella 9*	0.38	0.044	0.439	ADG
ASV69	Bacteria	F_Ruminococcaceae *UCG-014*	0.41	0.028	0.397	ADG
ASV82	Bacteria	Roseburia	0.49	0.009	0.340	ADG
ASV126	Bacteria	O_Mollicutes	0.49	0.008	0.340	ADG
ASV207	Bacteria	F_Ruminococcaceae *UCG-002*	0.44	0.020	0.397	ADG
ASV366	Bacteria	F_Ruminococcaceae *UCG-013*	0.45	0.018	0.397	ADG
ASV633	Bacteria	F_Ruminococcaceae *UCG-010*	0.41	0.030	0.397	ADG
Epithelial						
ASV10	Bacteria	F_Lachnospiraceae	0.45	0.021	0.356	ADG
ASV25	Archaea	Methanosphaera	0.44	0.024	0.096	ADG
ASV28	Bacteria	F_Prevotellaceae	− 0.43	0.028	0.356	ADG
ASV32	Bacteria	Prevotella_9	0.48	0.013	0.356	ADG
ASV69	Bacteria	Ruminococcaceae *UCG-014*	0.41	0.036	0.391	ADG
ASV181	Bacteria	O_Mollicutes *RF39*	0.46	0.017	0.356	ADG
ASV210	Bacteria	Mogibacterium	− 0.41	0.039	0.391	ADG
ASV366	Bacteria	F_Ruminococcaceae *UCG-013*	0.49	0.012	0.356	ADG
ASV426	Bacteria	F_Ruminococcaceae *UCG-009*	0.47	0.016	0.356	ADG
ASV912	Bacteria	F_Christensenellaceae	− 0.43	0.027	0.356	ADG

## Discussion

Prior to the completion of this study, the effects of breed on the microbial composition of the rumen liquid, solid and epitheliuem fractions in hill sheep, were unknown. However, recent studies in cattle have demonstrated that microbial taxonomic profiles vary between breeds, where the abundance of particular microbial species are regulated by host genetics^27^. As the rumen the rumen is comprised of 3 interconnecting microbial ecosystems; solid-, liquid- and epithelial ruminal fractions, we investigated (1) the effect of sheep breed on bacterial and archaeal populations in all three ruminal fractions, and (2) the effect of ruminal fraction on those populations in three breeds of sheep (i.e. Cheviot, Lanark and Perth). Our results provide the first report that diversity and abundance of bacterial and archaeal taxa in the solid, liquid and epithelial rumen fractions of sheep are influenced by breed. Our results expand and reinforce previous research in cattle showing differences in bacteria populations between breeds and ruminal fractions.

In the current study, breed was found to influence important production traits related to host feed efficiency, including FCR and ADG. Cheviot lambs were found to have the lowest mean FCR, indicating that it was the most feed efficient breed. However, the difference was only significant when compared to the Connemara breed, which had the highest mean FCR. Additionally, the Cheviot breed also had the fastest maturing lambs in the study, with 80% of lambs reaching maturity (> 40 kg) within the first 42 days of the study, with a mean LW of 47.1 kg. Among the SB strains the Perth had the lowest FCR. No differences in FCR and ADG between the Cheviot, Lanark and Perth were found. Although this is the first study to compare FCR and ADG between these mountain/hill sheep breeds, the findings are in line with a previous study that found that metabolic differences between six British sheep breeds (i.e. SB, Welsh Mountain, Cheviot, Suffolk Down, Kent, and Hampshire Down) were mostly similar^11^. However, when subjected to environmental stresses such as wind and rain, differences were apparent, with the SB found to more stress-tolerant than the Cheviot^11^.

Metagenomic studies investigating host genetic effects on the rumen microbiota have to date been performed using original rumen digesta^25,28^, which comprises both the liquid and solid ruminal fractions. The purpose of this study was to investigate the influence of sheep breed on bacteria associated with each of the three fractions independently. Our findings demonstrate that breed contributed to significant variations in alpha diversity (i.e. observed ASV’s and PD) in the solid, but not in the liquid, ruminal fraction. The solid fraction the Cheviot breed harbored a bacterial community that was less rich and more phylogenetically related than those of the SB, which was significant when compared to the Lanark breed. Previously, lower rumen microbial alpha diversity and richness were linked to higher feed efficiency in cattle^22^. An efficient rumen microbiome is considered to be less diverse and more specialised in metabolizing feed and delivering energy to the host^22^, which could be a factor influencing the greater FCR observed for the Cheviot breed in the current study. Our beta diversity analysis showed that bacterial communities associated with the liquid and solid fractions were not affected by breed, suggesting a large overlap of community representatives among breeds. Taken together our findings on bacteria diversity and composition contrast with an analogous study conducted in cattle^25^. Li et al.^25^ reported no significant differences in alpha diversity among three breeds of cattle; while PCoA based on Bray Curtis distances revealed that the Kinsella Hybrid breed exhibited a distinct bacteria community composition to that of the Angus and Charlaois breeds used in the study^25^. Conversely, an earlier study in cattle found both alpha and beta diversities differing between Holstein and Jersey cows^28^. Variations in community composition and diversity may be attributed to differences of animal model, management practice, diet, environment, age or analytical approaches used. We consider that a combination of these factors might explain differences between the current study and those studies mentioned. Although no major differences in bacteria community composition were observed, the abundance of several taxonomic groups were affected by breed in the solid ruminal fraction: *Sharpea* at the genus level, and *Sharpea azabuensis* and an unclassified ASV belonging to the family *Lachnospiraceae* at the species level. The Perth breed exhibited highest abundance of *Sharpea azabuensis* which was significant in comparison to the Cheviot breed. *Sharpea azabuensis* is a strictly anaerobic gram-positive bacterium that can metabolise a variety of sugars including d-glucose, d-fructose, d-galactose and sucrose producing lactate as the primary end product^29^. Previous studies investigating the rumen microbiota of sheep divergent for methane emissions have reported an enrichment of *Sharpea azabuensis* in lower methane emitting cohorts^30,31^. As a result, we suspect that the greater abundance of *Sharpea azabuensis* in the Perth breed may be suggestive of lower methane production, however, due to the lack of methane emissions data recorded in this study, this should be considered with caution. *Acetitomaculum* was identified as a dominant bacterial genus in the liquid ruminal fraction, with a mean relative abundance of 3.5%. Its abundance was found to be negatively correlated with FCR, indicating a potential role in enhancing host feed efficiency. Indeed, the abundance of an unclassified ASV within the genus (ASV44) was found to be higher in feed efficient Cheviots, which was significant when compared to the Connemara breed. *Acetitomaculum* has one known species, *A. ruminis*, an acetogenic bacterium capable of heterotrophic and autotrophic growth^32^. It possible that its higher abundance may be contributing to Cheviot feed efficiency by shifting H_2_ away from methanogenesis and towards acetogenesis, reducing dietary energy loss^33^. Alternatively, *Acetitomaculum* may be contributing to the improved FCR in the Cheviots through metabolic pathways other than reductive acetogenesis due to the organism's ability to metabolise a wide range of substrates and its inability to compete with methanogens for H_2_, especially at low H_2_ concentrations^32^.

Bacteria associated with the rumen epithelium maintain close interactions with the host and have been shown to correlate with ruminal epithelial tissue gene expression^34,35^, suggesting that host genetics may influence this bacterial population more than those in the solid and liquid fractions. In the current study, sheep breed was found to significantly contribute to differences in the alpha diversity (i.e. observed ASVs and inverse Simpson), but not beta diversity of the epithelial-associated bacterial community. In the context of alpha diversity, the Cheviot breed harbored the fewest number of observable ASVs, while the Connemara harbored the most. Moreover, when compared to Connemara and Perth, the Cheviot and Lanark breeds had a significantly higher mean inverse Simpson index. This finding suggests that, while epithelial community richness was lowest for the Cheviot breed, the community was more uniformly distributed with respect to species abundance than those of the Connemara and Perth breeds. Firmicutes, Bacteroidetes, and Proteobacteria were the most predominant bacterial phyla, which is consistent with previous studies exploring epithelial bacterial communities in cattle^36,37^*.* ASV24 classified to the genus *Succiniclasticum* was shown to be more abundant in the epithelia of the Cheviot, Lanark, and Perth breeds when compared to the Connemara breed. *Succiniclasticum* is a gram-negative rod-shaped anaerobe that ferments succinate and converts it to propionate^38^, an important precursor of glucose in the rumen^39^. The higher abundance of *Succiniclasticum* in those breeds may have contributed to their enhanced FCR compared to the Connemara breed by supplying enough extra propionate to boost gluconeogenesis, which is important for animal growth and production^40^. The abundance of *Ruminococcus 1* was significantly higher in the Perth and Connemara breeds relative to the Cheviot breed. *Ruminococcus* spp. are core members of the rumen microbiome^41^, and its association with the rumen epithelial could indicate that its abundance is under host genetic regulation. Indeed, previous research carried out by Li et al. showed *Ruminococcus* was heritable in cattle (h2 = 0.16 ± 0.08; mean ± SE), and variations in its abundance were associated with a single nucleotide polymorphism (SNP) in the RAPH1 gene^27^. The genus comprises some of the most proficient and best described cellulolytic degraders, including *R. albus* and *R. flavefacians*^42^. Consequently, it is probable that the Connemara and Perth breed are genetically selecting for a higher abundance of *Ruminococcus*, which may have allowed these SB breeds to evolve into successful mountain sheep able to thrive in poor grazing areas with low-energy vegetation.

Bacterial community profiles can differ across ruminal fractions in both bovine and ovine ruminants^14,16^. Therefore, we investigated the influence of ruminal fraction on bacterial populations in the Cheviot, Connemara, and Perth breeds individually. Our findings show that ruminal fraction had no effect of alpha diversity measures in the Cheviot breed. However, in the Lanark breed the liquid fraction exhibited a significantly lower Shannon diversity, while in the Perth breed the epithelial fraction exhibited a significantly higher community richness and PD when compared to the other fractions, respectively. It is widely reported in the literature that the bacterial community composition of the epithelial fraction is distinct from those communities associated with rumen content^14,16,43^. In contrast to prior studies^16,18^, our beta diversity analysis revealed no significant differences in community composition across ruminal fractions for any of the breeds studied. The reason for this finding is unclear, though it could be related to variations in dietary management between studies^44–46^. While no major compositional differences were seen in this study, ruminal fraction had a significant impact on the abundance of taxa commonly associated with the rumen epithelium, with the majority of differences occurring between the epithelial and solid fractions in all breeds. When considering the Lanark and Cheviot breeds, several taxa within the order Clostridiales (Family XII*, Butyrivibrio 2, Mogibacterium* and Lachnospiraceae *UCG-008*) were significantly more abundant in the epithelial ruminal fraction. Clostridiales are obligate anaerobes that have previously been discovered to interact with the rumen epithelium^47,48^ and reported to be key components of its core microbiota^45^. In addition to Clostridiales, *Campylobacter* was also found to be significantly abundant in the epithelial fraction of the Cheviot and Lanark breeds. *Campylobacter* is an asaccharolytic microaerophilic bacterium that has frequently been identified as associating with the rumen epithelium^16,49,50^, and its capacity to consume oxygen demonstrates its functional significance in maintaining the rumen's anaerobic environment^51^.

Archaea are the sole producers of methane within the rumen, which is an important homeostatic process that regulates the partial pressure of hydrogen^52^. However, methanogenesis is estimated to result in a 2–12% loss in feed efficiency to the host^53^, and is further supported by research that has revealed associations between ruminant methane emissions and host feed efficiency^54^. Moreover, the abundance of methanogenic archaea in the rumen has been linked to both methane emissions^55^ and feed efficiency^56^. To date, research in cattle has shown that archaea taxonomic abundances vary between breeds of cattle^25^ and some archaeal species have been found to be heritable^27,57^, signalling a potential host genetic effect on the community. Therefore, in the present study we explored the effect of sheep breed on archaeal populations associated with the solid, liquid and epithelial fractions. Alpha diversity analysis showed that breed had a significant effect on the richness of the solid and liquid associated communities, whereby the Cheviot breed exhibited the lowest community richness. Beta diversity analysis revealed no significant effect of breed on community composition, suggesting the presence of a shared core archaea community among breeds. Similarly, differential abundance analysis revealed no overall influence of breed on taxonomic abundances; however, pairwise comparison between breeds shows that the genus *Candidatus Methanomethylophilus* was significantly more abundant in Perth and Lanark breeds compared to the Cheviot breed in the solid ruminal fraction. Furthermore, results from our correlation analysis showed *Methanosphaera* and *Candidatus Methanomethylophilus* associated with improved ADG and feed efficiency in the epithelial and liquid ruminal fractions, respectively. *Candidatus Methanomethylophilus* is a H_2_-dependent methylotrophic methanogen, which derives its energy from the metabolism of methanol and methylaimines^58,59^ In our previous study with sheep, *Candidatus Methanomethylophilus* was identified in both the solid and liquid ruminal fractions, but significant correlation was not observed between its abundance and FCR^21^. In contrast, Li et al. (2019) found the abundance of *Candidatus Methanomethylophilus* significantly higher in H-RFI Charlois steers when compared to L-RFI counterparts, however, similar findings were not observed in the Angus or Hybrid Kinsella breeds used in that same study^25^. Furthermore, *Methanosphaera* and *Candidatus Methanomethylophilus* have previously been linked to lower methane emitting in sheep and cattle^31,60^, respectively. However, given the complexity of methane synthesis in the rumen, associating higher or lower methane production to individual taxonomic groups may be unrealistic^61,62^.

The results in the current study further support findings in the literature that breed/host genetics can influence the microbial community structure within the rumen. This could have applications for breeding programs, where microbiomes that are better at utilizing feed and producing less methane could potentially be selected for^63,64^. However, the heritability of rumen microbiome composition across generations needs further investigation in livestock ruminants, including with microbiome transplant experiments. Because of the functional redundancy of the rumen microbiome^65^, where phylogenetically distant microbes may have identical metabolic capabilities, taxonomic differences observed between breeds in the current study may not necessarily reflect functional divergence. Future research would benefit from coupling microbial community composition with rumen chemistry, in addition to multi-omics approaches (i.e. meta-genomics/transcriptomics), which would give a better indication of the rumen microbiota's varied metabolic capacity between sheep breeds.

## Conclusions

In summary, this study demonstrated the breed of sheep has an effect on the bacterial and archaea taxonomic abundance within the rumen, which can have significant implications for improving feed efficiency and reducing methane emissions. However, further research is required to determine if the taxonomic differences observed signifies functional variation between the breeds. Furthermore, we observed differences in the distribution of bacterial taxa between ruminal fractions, which supports previous studies and highlights a rumen fraction bias of importance to rumen sampling strategies for rumen sampling strategies.

## Methods

### Animal model

The Teagasc Animal Ethics Committee authorised all treatments involving animals in this investigation, which was conducted under experimental licence (No:P19132/P028) from Ireland’s Health Product Regulatory Authority (HPRA) in compliance with ARRIVE guidelines and the European Union protection of animals used for scientific purposes regulations 2012 (S.I. No 543 of 2012).

Over a 3-month period, data was collected on 36 ram lambs enrolled in a feed efficiency measurement test. Lambs included in this study originated from four different breeds: Cheviot (n = 10), Connemara (n = 6), Lanark (n = 10) and Perth (n = 10). After weaning, lambs were individually penned on plastic slat-floored feeding pens (182 cm L × 122 cm W). Lambs were allowed tactile, olfactory, and visual contact with each other through the pen partitions. The mean body weight of animals at the beginning of the measurement period was 29.6 kg (SD = 3.7). Throughout the trial period, all lambs were offered a cereal-based nut ad libitum, with fresh concentrates supplied daily and refusals removed (i.e., troughs emptied and cleaned) weekly. Concentrates were weighed daily in the morning, and daily intake was estimated by subtracting the weekly total intake from the number of refusals and dividing by seven. Concentrates were supplemented with unrestricted access to perennial rye-grass silage (*Lolium perenne*) to maintain rumen health (100-g/d DM). Silage was offered fresh daily and refusals removed twice weekly during morning feeding. At no point were animals without access to concentrates or silage during the ad libitum feeding period. Silage intake was not measured as consumption was low. Table 11 contains the ingredients and chemical composition of concentrate and silage used in the study. At all times throughout the measurement period lambs had access to fresh drinking water. The feed intake measurement period ceased when lambs reached a target slaughter weight of > 40 kg. Lambs were slaughtered at the Kepak Ltd abattoir in Athleague in Co. Roscommon on three separate dates when lamb maturity was reached; 29th November 2017, 13th December 2017 and 17th January 2018. The abattoir was approximately 56 km (55 min) from the Teagasc research farm in Athenry, Co. Galway, Ireland. Prior to slaughter, feed and water (at the farm) were withheld for 2 h for all sheep in the study, since differences in time off feed may have affected rumen microbial community composition.

**Table 11:** Ingredient and chemical composition of concentrate and silage offered to lambs.

Concentrate		Silage
Ingredient (kg/tonne)		
Maize	300	–
Barley	300	–
Soya hulls	165	–
Soya bean meal	155	–
Molasses	50	–
Minerals	30	–
Chemical composition		
DM, g/kg	850	255
DMD	**–**	740
Composition of DM, g/kg		
CP	172	133
NDF	278	642
ADF	145	364
Ash	62	100

DMD dry matter digestibility.

Phenotypic data collected throughout the trial period included the animals weight at the beginning of the trial period (Start weight); dry matter intake (DMI), described as the amount of feed (kg) the lambs consumed; average daily gain (ADG) was calculated by dividing the total weight gain over the trial period divided by the number of days animals were on trial before slaughter; feed conversion ratio (FCR) was calculated by dividing DMI by ADG. Live weight (LW) was the weight of lambs before slaughter. LW gain was the difference in weight at the beginning and end of the trial period. Carcass weight refers to the weight of the carcass after the offal has been removed following slaughter. KO% refers to the weight of the carcass as a percentage of the animal's live weight prior to slaughter.

### Sample collection

Samples of ruminal fractions were collected immediately after slaughter. Rumen fluid and solid fractions were separated into 25 ml tubes, by filtering rumen digesta through four layers of sterile cheesecloth. To collect rumen epithelial samples, papillae were cut from dorsal, ventral, cariad and caudal regions of the rumen wall using sterilised scissors, approx. 1 cm^2^, and subsequently rinsed with cold sterile saline solution (0.9% w/v NaCl). Samples from all three ruminal fractions were frozen immediately in liquid nitrogen after separation and then stored at − 80 °C. A total of 90 samples were available for the current study, 28 epithelial samples (Cheviot n = 9, Connemara n = 3, Lanark n = 7, Perth n = 9), 30 liquid (Cheviot n = 9, Connemara n = 5, Lanark n = 9, Perth n = 7), and 32 solid ruminal samples (Cheviot n = 8, Connemara n = 6, Lanark n = 9, Perth n = 9).

### Rumen microbial DNA extraction and library preparation

Under liquid nitrogen, each sample was homogenised to a fine frozen powder using a pestle and mortar. Extraction of microbial DNA from the samples was performed using the method described by Yu and Morrison^66^. Amplicon libraries were created from 25 ng of rumen microbial DNA using two rounds of PCR amplification as described in the Illumina Miseq 16S Sample Preparation Guide, with minor alterations to cycle length as described by McGovern et al.^17^. 515F/806R primers^67^, built with Nextera over hang adapters, and 2× KAPA Hifi HotStart ReadyMix DNA polymerase were used for the first round of PCR amplification, targeting the V4 hyper-variable region of the 16S rDNA (Roche Diagnositics, West Sussex, UK). The first round of PCR was performed at 95 °C for 3 min, then 20 cycles of 95 °C for 30 s, 55 °C for 30 s, 72 °C for 30 s, and 72 °C for 5 min. To enable the attachment of dual indices and Illumina sequencing adapters using the Nextera XT indexing kit, a second round of PCR was conducted at 95 °C for 3 min, followed by 8 cycles at 95 °C for 30 s, 55 °C for 30 s, 72 °C for 30 s, and 72 °C for 5 min (Illumina, San Diego, CA, USA). Following PCR rounds 1 and 2, the amplicons were purified using the Qiaquick PCR Purification Kit (Qiagen, Manchester, UK). To remove adaptor primers, amplicons were pooled together in identical concentrations and gel purified using the Qiagen Gel Extraction Kit (Qiagen, Manchester, UK). Using the QIAquick PCR purification kit, the amplicons were again purified to eliminate any agarose residues (Qiagen, Manchester, UK). Amplicon purity was measured using the Nanodrop 1000, followed by confirmation using the Qubit fluorometer and the KAPA SYBR FAST universal kit with Illumina Primer Premix (Roche Diagnositics, West Sussex, UK). Amplicon libraries were diluted and denatured according to the Illumina Miseq 16S Sample Preparation Guide, and sequencing was performed on the Illumina MiSeq using the 500 cycle version 2 MiSeq reagent kit (Illumina, San Diego, CA, USA).

### Bioinformatics

Amplicon reads were quality assessed using FASTQC (version 0.11.5)^68^. Adapters and ambiguous basecalls were subsequently removed using Cutadapt (version 1.18)^69^. The amplicon reads were processed and analyzed using the Divisive Amplicon Denoising Algorithm 2 (DADA2) (DADA2)(version 1.18.0), as described in Callahan et al.^70^. The DADA2 tutorial available at https://benjjneb.github.io/dada2/tutorial.html (version 1.12) was followed for read filtering, dereplication, sample inference, chimera elimination, paired end read merging, and taxonomy categorization. Taxonomic classification was performed to the species level using the SILVA classification databases (version 132)^71^. The final output from DADA2 was an Amplicon Sequence Variant (ASV) table and a corresponding taxonomy table. A phylogenetic tree was created using the R package Phangorn^72^. Prior to downstream analysis, a Phyloseq object including the ASV table, taxonomy table, phylogenetic tree, and experimental metadata was created using the R/Bioconductor package Phyloseq (version 1.26)^73^. Six samples (2 solid, 2 liquid and 2 epithelial) were removed prior to downstream analysis as a result of having a substantially reduced number of reads (< 100 reads).

### Compositional and statistical analysis

Animal production trait data were first verified for normality and homogeneity using the Shapiro Wilks and Levenes tests, respectively, and then compared among breeds using a two-way ANOVA followed by a *post-hoc* Tukey HSD. DMI records were missing from one animal in each of the Connemara, Perth and Lanark breeds, as a result those animals were omitted from DMI and FCR comparisons.

To profile dominant bacterial and archaea taxa, raw counts were converted to relative abundances and the mean and standard deviation relative abundance of dominant phyla and genera were reported. To examine the effect of breed on bacterial and archaeal populations the data was first stratified according to ruminal fraction (i.e. solid, liquid and epithelial) and then compared between breeds (i.e. Cheviot, Connemara, Lanark and Perth). Similarly, to examine the effect of ruminal fraction on microbial profiles data was stratified according to breed and compared between fractions. For fraction analysis only animals where all three ruminal fraction were available were considered. Due to low numbers of biological replicates the Connemara breed was excluded from ruminal fractions analysis. Prior to diversity analysis raw counts were normalised to even sampling depth using the scaling with ranked subsampling (SRS) method^74^ and rarefaction curves were generated to assess sequencing effort. Following assessment of rarefaction curves, sample 2083-Solid, which had a sequencing depth of 13,922, was removed due to a loss in community diversity when rarefying. Shannon, inverse Simpson, Faiths phylogenetic diversity (PD), and observed ASVs (richness) diversity indices were used to generate within-sample (alpha) diversity metrics. Alpha diversity data were checked for normality and homogeneity using Shapiro Wilks test and Levenes test prior to statistical analysis. A two-way ANOVA was used to test the null hypothesis that no difference on mean alpha diversity measures existed between groups. For beta diversity analysis, dissimilarities in community composition were measured using both weighted and unweighted UniFrac distances and visualised using principle coordinate analysis (PCoA). Differences in community composition was tested with PERMANOVA and conducted with 9999 permutations using the Adonis function from the R/Bioconductor package Vegan (version 2.5-5)^75^. Differential abundance (DA) analysis was conducted using both the likelihood ratio test (LRT) and the Wald’s test from the DESeq2 package^76^. Only taxa with a relative abundance larger than 0.01 percent and a prevalence greater than 50 percent were considered for DA analysis. An a priori q-value threshold was set at 0.05. The date of sample collection was included as a covariate to adjust for variations in abundance associated with different slaughter dates. Lastly, Spearman correlation coefficients (*P* < 0.05) were done to assess the correlation between bacterial and archaeal genera and animal production traits; FCR and ADG, in order to identify potential feed efficiency drivers. Only genera with a relative abundance larger than 0.01 percent and a prevalence greater than 50 percent were considered for correlation analysis.

### Ethics declaration

Irelands Health Products Regulatory Authority reviewed and authorised the animal study in compliance with the European Union (EU) protection of animals used for scientific purposes regulations 2012 (S.I No 543 of 2012).

## Data Availability

Data used for this study were deposited into NCBI Sequence Read Archieve (SRA), published under accession number PRJNA781265.
